# What makes rhythms hard to perform? An investigation using Steve Reich’s Clapping Music

**DOI:** 10.1371/journal.pone.0205847

**Published:** 2018-10-18

**Authors:** Sam Duffy, Marcus Pearce

**Affiliations:** Music Cognition Lab, Queen Mary University of London, London, United Kingdom; University College London, UNITED KINGDOM

## Abstract

*Clapping Music* is a minimalist work by Steve Reich based on twelve phased variations of a rhythmic pattern. It has been reimagined as a game-based mobile application, designed with a dual purpose. First, to introduce new audiences to the Minimalist genre through interaction with the piece presented as an engaging game. Second, to use large-scale data collection within the app to address research questions about the factors determining rhythm production performance. The twelve patterns can be differentiated using existing theories of rhythmic complexity. Using performance indicators from the game such as tap accuracy we can determine which patterns players found most challenging and so assess hypotheses from theoretical models with empirical evidence. The app has been downloaded over 140,000 times since the launch in July 2015, and over 46 million rows of gameplay data have been collected, requiring a *big data* approach to analysis. The results shed light on the rhythmic factors contributing to performance difficulty and show that the effect of making a transition from one pattern to the next is as significant, in terms of pattern difficulty, as the inherent complexity of the pattern itself. Challenges that arose in applying this novel approach are discussed.

## Introduction

Rhythm can be perceived and reproduced, both in music and in our wider environment. We are adept at recognising regular rhythmic patterns, for example the sound of train carriage wheels passing over track joins, a heartbeat, a tap dripping or a clock ticking. We synchronise with rhythms (a phenomenon known as entrainment) consciously and unconsciously, for example tapping our feet as we listen to music. We respond to the regularities that are present in our auditory environment, inferring hierarchical patterns of regularly accented pulses, known as *metre* [1]. Some pulses are perceived as more accented than others and perception of such accents can depend on the structure of the rhythm, the arrangement of the notes and rests, or inferred from the volume, timing, articulation, intonation and timbre of a note. Accents can be produced expressively, the performers communicating their feel for the metre through the way that they articulate each note. They can also arise in mechanically reproduced rhythms, a simple example being a metronome in which a different sound is used to distinguish the pulse at the beginning of each repeated bar. Some rhythms may be perceived as simple, whilst others are perceived as more complex and are harder to entrain to or reproduce. For example metrical rhythmic patterns, with integer ratio relationships between pulse intervals and regular perceptual accents, are easier to reproduce than non-metrical patterns [2, 3].

Rhythmic coordination of perception and action has been studied in many contexts, and has particular relevance for musical performance, but research tends to focus on finger tapping in response to auditory stimuli rather than materials that approach the complexity of real music [4, 5]. Laboratory based experiments are valuable, but also limited by the number and range of participants that can take part. What if, instead of bringing participants to the laboratory, we made the study available to them in their environment? What if we made the test part of a compelling activity, embedded in real music, which encouraged repeated engagement? Whilst we may lose some of the experimental control available in the psychology laboratory, we gain in terms of the number of people who can take part, and the variety of participants in terms of musical training and sophistication, cultural background, geographic location, age and education, which are important sources of bias in much of the behavioural sciences [6].

This approach follows a newly developed methodology in the psychological and cognitive sciences that takes advantage of the ubiquity of mobile computers (smartphones and tablets) and online collection of large amounts of user data. Brown et al. [7], presented four classic experimental paradigms from the psychological literature as short games in a free app and found that the large sample size (20,800 users in one month) vastly outweighed the noise inherent in collecting data outside of a controlled laboratory setting. Griffiths [8] laments the predominant use of large databases of online behaviour for purely behavioural analysis, an example being recommendation of music based on shared listening patterns with other users (e.g., “people who listened to this artist also listened to this other artist”, also known as *collaborative filtering* [9]). He calls instead for a revolution that uses such databases to evaluate models of human cognition, which requires the use of theoretical cognitive principles as a bridge between:

1. lab studies that are small in scale and narrow in scope but rigorously controlled; and
2. online studies that are large in scale and broad in scope, but noisy and uncontrolled, which can make it difficult to establish causality.

The present research is motivated to do exactly this, combining several cognitive theories of rhythmic complexity derived from previous lab-based studies, in order to investigate performance accuracy of a real piece of music, as performed by tens of thousands of people worldwide on their own mobile devices.

Specifically, we use a game-based application for iPhone and iPad based on the piece *Clapping Music* by Steve Reich to investigate the influence of rhythmic complexity on performance. Downloaded over 100,000 times in over ninety countries during a one year period, the app provides an opportunity to understand how people assimilate and reproduce twelve different rhythmic patterns in the context of a real piece of music, performed ‘in the wild’ so to speak. Having collected over 30GB of gameplay data at a detailed level, the analysis of the data becomes a *big data* challenge. Whilst there are problems associated with collecting and analysing such a large dataset, it presents a unique opportunity to understand which aspects of performing the piece Clapping Music present the most difficulty.

The paper is organised as follows. In the remainder of the Introduction, we introduce Clapping Music as a piece of music and the game included within the app, review previous relevant literature on rhythm and metre perception and consider in detail the implications of existing theories of rhythmic and metrical complexity for performance of Clapping Music. We then provide the Methods used to collect the data before presenting the results in five sections: first, descriptive properties of the dataset; second, examining evidence that users were motivated to complete the game; third, using tap accuracy as a measure of difficulty for the different rhythmic patterns in Clapping Music; fourth, examining the difficulty of transitions between patterns; and fifth, examining pattern difficulty with the transition effect removed. Finally, the results are discussed and limitations and future directions are presented.

### Clapping Music: The piece

**Fig 1:**
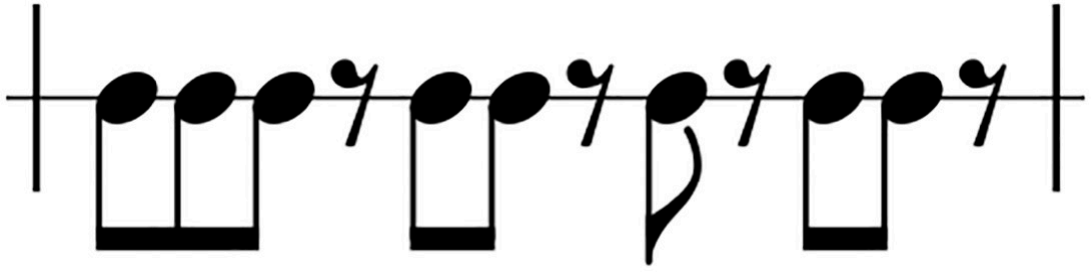
The rhythmic pattern that forms the basis of Steve Reich’s Clapping Music.

The app is based on a contemporary piece of music by the minimalist composer Steve Reich. Phasing is used to transform a short rhythmic pattern into twelve variations that form an ensemble piece, performed by two people clapping. The pattern is based on 12 note events, represented in the score as a combination of 8 quavers and 4 quaver rests (Fig 1). The phasing is discrete, the pattern being transformed one quaver at a time, in contrast to Reich’s earlier pieces such as Piano Phase, where the phasing is continuous. This is a deliberate change:

“Clapping Music marks the end of my use of the gradual phase shift process…I felt a need to find new techniques.”

[10]

**Fig 2:**
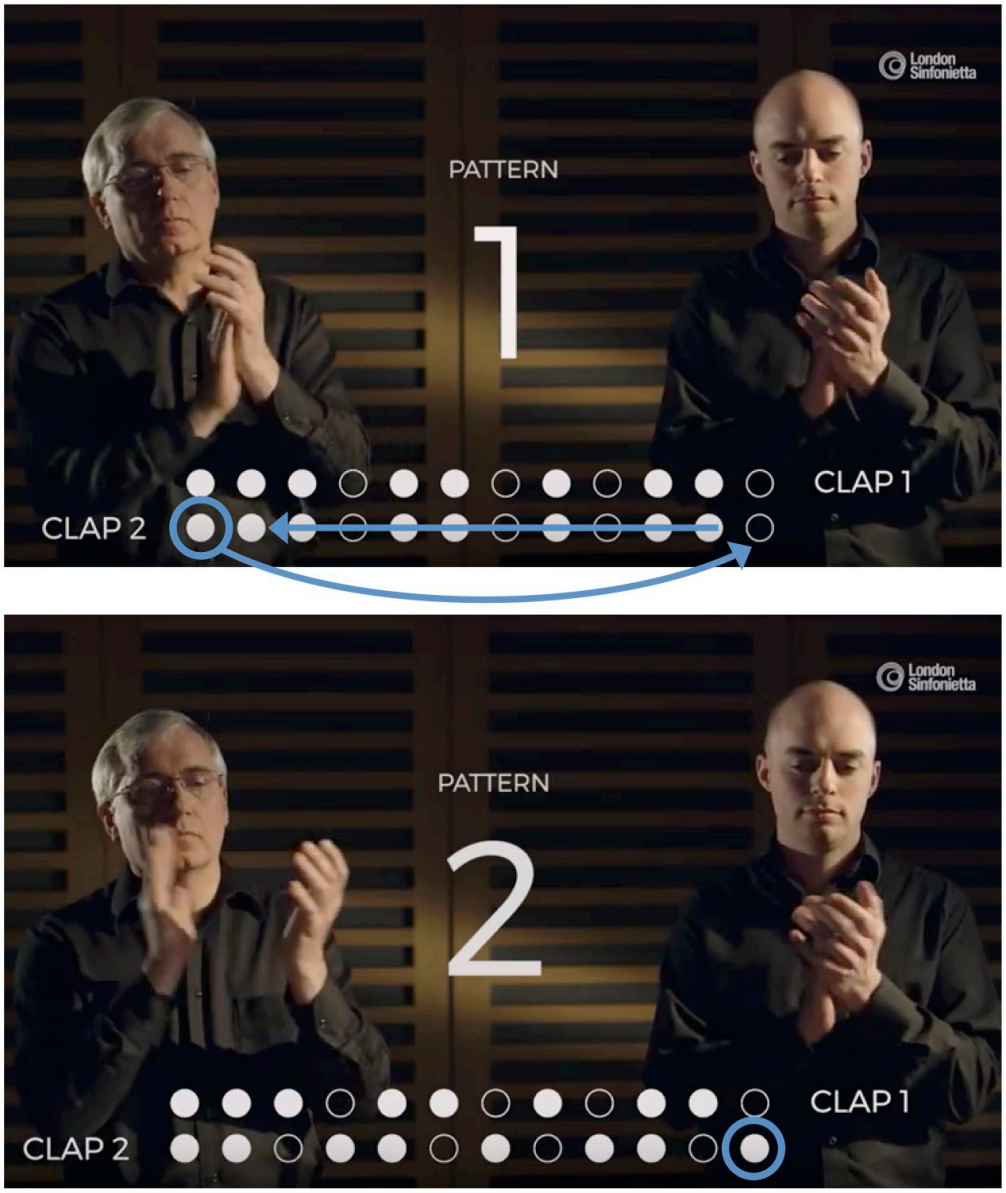
Transforming the static pattern to form the first variation. The blue arrows show how pattern 1 is transformed into pattern 2, by moving the first note to the end of the row and shifting the rest of the pattern forward. Source: A still from a performance film featuring David Hockings and Toby Kearney, produced by The London Sinfonietta exclusively for the App.

**Fig 3:**
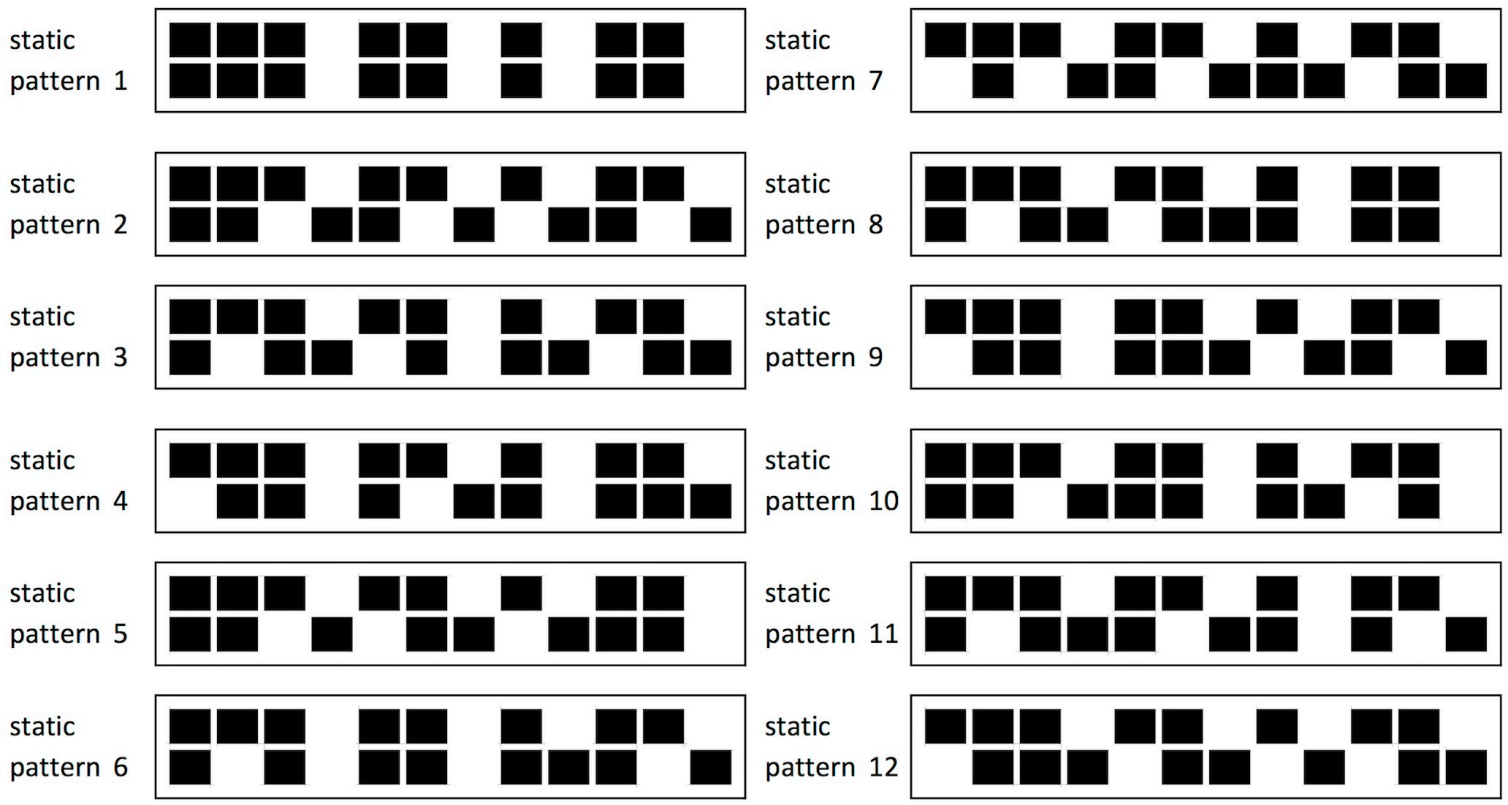
The twelve pattern combinations in Steve Reich’s Clapping Music.

The piece begins with the performers clapping the pattern in unison. After a number of repetitions (playing each pattern 12 times is recommended in the score) one person shifts the pattern by moving one beat from the beginning of the pattern to the end (the *phasing pattern*, Fig 2), whilst the other person continues playing the pattern in its original form (the *static pattern*). This results in the two forms of the pattern being played together at the same time, but out of phase by one quaver. After twelve more repetitions the person who transformed the pattern shifts it again, moving another beat from the beginning of the pattern and adding it to the end, whilst the person performing the static pattern continues to play the original form. There are 12 pattern combinations in total (Fig 3), each with a unique feel, for example some patterns begin with a rest and some have more notes in unison with the static than others. Once the phasing pattern has been shifted twelve times it returns to being aligned with the static pattern, and the performers play in unison to complete the piece. Each performer has a unique rhythmic challenge. One has responsibility for maintaining a constant pulse through repetition of the static pattern, without being influenced by the different pattern variations being played against it. The other is responsible for making smooth transitions between the patterns, keeping in time with the pulse of the static pattern.

### Clapping Music: The game

Steve Reich’s Clapping Music App (‘the App’) is a digital application including a game, videos and other content related to the music of Steve Reich and the music genre Minimalism, which is free to download from the iTunes Store for Apple devices running iOS 8 and above (from https://itunes.apple.com/app/id946487211). The App was developed through an interdisciplinary collaboration between The London Sinfonietta, a world-leading orchestra in the field of contemporary classical music, Touchpress, developers of apps for Apple iOS devices and the Music Cognition Lab at Queen Mary University of London (see [11]). There have been over 140,000 downloads worldwide since the launch on 9th July 2015.

In the App, the device takes the part of the performer playing the static pattern and the player of the game takes the part of the performer making the pattern transitions. The game was designed so that you do not need musical training to play, for example representing quaver beats and rests through full or empty circles rather than musical notation. Rather than clapping, players tap in a performance area in the lower part of the screen. This was due to considerations of latency related to device microphones, and the need to isolate the clap from other sounds if the game was played in a noisy environment. Tapping also enables the game to be played using headphones without adding noise to the environment, maximising playing opportunities. Tapping, like clapping, is a discrete movement with tactile feedback but the absence of auditory feedback could make it more difficult to synchronise with the static pattern. A study investigating the role of movement in synchronisation to music found that participants were less able to synchronise with musical stimuli through bouncing than clapping, perhaps due to the absence of auditory and tactile feedback [12]. As a result, in the App each tap is represented audibly by a sampled clap sound so that the player can hear their tapping against the sound of the static pattern and get a feel for the ensemble performance. Clap sounds were sampled from the performance recording of David Hockings and Toby Kearney that appeared in the App (see Fig 2). McAdams et al. [13] describe a streaming effect, where two different patterns played simultaneously cannot be perceived separately, under certain conditions (see also [14]). The performance directions in the Clapping Music score indicate that this is a desirable effect:

“Whichever timbre is chosen, both performers should try and get the same one so that their two parts will blend to produce one overall resulting pattern.”

[15]

However, player feedback from prototype testing of the App suggested that the game would be easier if there was some perceptual difference between player taps and the static pattern. The static pattern is created from one of David Hocking’s claps, the player’s tap is represented audibly by one of Toby Kearney’s claps. A player may audibly distinguish between the sound produced by their taps and the static pattern as the timbre is slightly different.

**Fig 4:**
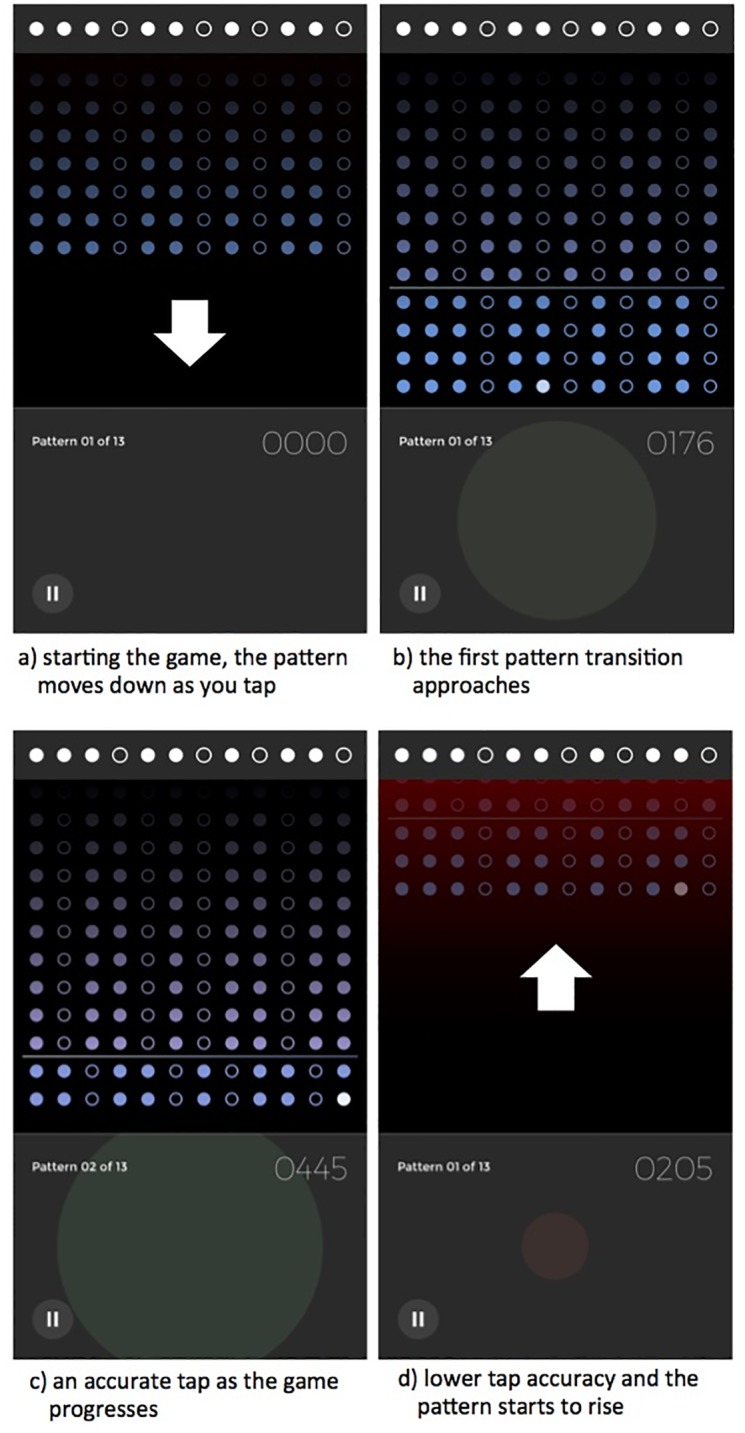
Playing the game in Steve Reich’s Clapping Music App.

The accuracy of tapping is determined algorithmically. The target rhythm is divided into time bins corresponding to intervals of a demisemiquaver (32nd note). Incoming performed taps are quantised to the nearest bin and then scored depending on temporal proximity to the nearest target bin. A tolerance can be set so that taps in bins either side of the target bin receive non-zero scores. The tolerance is greatest at the easy level of difficulty in the game but is reduced for the medium level, and reduced again for the hard level. Scores for individual taps are summed and normalised to yield a score for each completed pattern row (or loop) ranging between 0.00 (the lowest possible accuracy) and 1.00 (perfectly corresponds to the target pattern). If the player does not tap the correct number of claps in any given pattern, or does not clap for more than two beats, maximum error is recorded for that row. The player’s accuracy is represented in the performance area—a large green tap area is good (Fig 4c), a small red tap area represents an inaccurate tap (Fig 4d). To add a further gaming element, if tap accuracy meets a target threshold then the pattern moves down the screen (Fig 4a) and the player is able to transition to the next pattern (Fig 4b). If accuracy is too low or inconsistent, then the pattern moves up the screen, a red background indicating that the player is in trouble (Fig 4d). The dots representing note events turn white as they are due to be played, to help players get back on track. If the player’s accuracy improves the pattern will start to move back down the screen again. The game is over when the pattern reaches the top of the screen or the player has managed to play all 12 patterns, finishing with a final unison repetition of the original pattern.

The first pattern needs to be played accurately for a minimum of 8 repetitions. The first transition will not be offered until the pattern dots reach the bottom of the screen, as a result of consistently accurate tapping. As a result, players could tap more than 8 loops before making their first transition. Once a successful transition has been made, a minimum of 6 repetitions is required for all of the following patterns. Again the number of loops played per pattern will be more than this if accuracy is not maintained. The player receives a score determined by their accuracy for each row of the pattern completed, and the number of transitions they have made.

There are three levels of difficulty in the game, which can be chosen by the player—easy, medium and hard, distinguished by tempo and the accuracy threshold. The accuracy thresholds for each level of difficulty were set using feedback from prototype testing. The easy level also has a metronome, which we will discuss later. There is a training area where any of the patterns can be practiced, and additional content including a full performance and an exclusive interview with Steve Reich, who has endorsed the app.

### Rhythm and beat perception

The representation of rhythmic structure, and how it is performed, influences how the listener might perceive it. It is useful to define some terms to describe the temporal organisation of music. While the word *rhythm* is used in a variety of ways, in its most specific form, a rhythm denotes a pattern of *inter-onset intervals* (IOIs) created between the temporal onsets of a sequence of events, which differ in the number of events and the temporal spacing between them. Each of the 12 patterns of the phasing part of Clapping Music constitute different rhythms. Under particular conditions, listeners may infer metrical structure (a *metre*), a hierarchy of stronger pulses, which are accented in some way when compared to weaker pulses, in a regular, repeating structure. If a pulse appears at a particular level, it also appears at the next, larger level, making metrical structure hierarchical [16]. So whilst pulse establishes a single periodicity, it is insufficient in itself to determine the accented groupings that are perceived as metre [1]. The most salient level in the metrical hierarchy is called the *tactus* or *beat*, the rate at which you might clap your hands, or tap your feet, as you listen. A higher level in the metrical structure is referred to as a *measure* or *bar* [17]. Time signatures are notational conventions used in Western music to indicate the metre intended by the composer as a direction to aid performance. The time signature 4/4 indicates that the *pulse* is a crotchet (or quarter note) and each bar contains 4 crotchets, which would normally be perceived as the tactus level. The time signature 12/8 indicates that the pulse is a quaver (or eighth note) and each bar contains 12 quavers (the tactus here would usually be felt every three quavers—a *compound time signature*). Tempo refers to the speed with which a rhythm is performed and is usually defined in terms of the rate of the tactus.

Each level in the metrical hierarchy defines regularly recurring subdivisions of time, with stronger metrical accents coinciding with higher levels in the metrical hierarchy. An auditory stimulus (such as a piece of music) implies a metre to the extent that salient events appear on strong metrical accents in the metre. Events may be salient by virtue of their loudness, timbre, pitch or temporal relationship with other events. Once inferred, listeners are relatively resistant to changing their metrical interpretation [18]. Perception of metre depends on performance characteristics, and the musical training and cultural background of the listener. Not all listeners infer metre in the same way. For example, in studies where participants were asked to tap along to a rhythm some tapped at the beat level, others at the measure level or the two-measure level [19]. Furthermore, listeners from different musical cultures may infer metrical structure in different ways due to incidental exposure to the presence or absence of particular metres in the music of those cultures [20–22].

Rhythmic skill can be assessed in a number of ways, for example the ability of an individual to reproduce a rhythm from memory, to distinguish rhythms with subtle variations or to synchronise with a pulse by reproducing a perceived beat. Some research suggests that listeners have an internal clock, and can perceive accents in a pattern purely from temporal (time based) information. The more temporally regular a music excerpt is, the easier it should be for the listener to extract the underlying beat [23]. According to this approach, beat extraction should be easier for mechanical performances, for example audio stimuli synthesised by a computer, than for real performances with expressive variations. The ability to reproduce a pattern is strengthened by providing an external clock, such as a metronome. An alternative hypothesis, which is more applicable to expressive performances, is that the performer constructs a mental representation of the metrical structure, which they convey through the performance microstructure and expressive variation, making perception of an underlying beat easier. The perceived beat in this case is influenced by the performer’s interpretation of the rhythmic structure [4, 5]. This implies that listeners, depending on the expressive content, may perceive the rhythmic structure of different performances of the same notated music differently.

A listener’s degree of musical training also influences the extent to which the expressive elements of performance of a rhythm are useful for beat perception. Drake et al. [19] compared musicians’ and non-musicians’ ability to tap in time with three variations of six excerpts of music; mechanically produced, mechanical with the first beat of the bar accented and an expressive performance. It was found that participants synchronized most successfully with the accented mechanical rhythm, followed by the mechanical version with no accent, and then the expressive version. In all three cases, musicians were better at synchronisation than non-musicians, but musical training did not change the extent of responsiveness to expressive compared to mechanical excerpts, which supports the existence of an internal clock. A study examining perception of rhythmic similarity between the patterns in Clapping Music reinforces this finding. It was found that expressive performance helped the non-musical participants more than the musically trained participants, perhaps due to the fact that musicians have experience in listening to, processing, and distinguishing rhythms as conceptual objects, rather than as purely auditory objects [24]. We will now examine how existing research on beat perception and rhythmic complexity can be applied to the design of the Clapping Music App and the analysis of the performance data that it generates.

### Beat perception in Clapping Music

The mechanical nature of the static pattern reproduced in the game means that it lacks expressive accents but players may still perceive a metre. The perceptual effects of a sound depend on the musical context in which that sound is embedded. A given sound’s perceived pitch, timbre, and loudness are influenced by the sounds that precede it, coincide with it, and even follow it in time [13]. These perceptual effects are not limited to expressive aspects such as relative beat volume or timbre, but can also be influenced by temporal effects such as the relative timing of note events in a measure. Cameron et al. [24] assessed the ability of individuals to perceive similarity between the 12 patterns in Clapping Music and found that when a rhythm with a large number of rests in common with the static pattern was heard after a rhythm with fewer rests in common, the pair was rated as less similar than when heard in the opposite order. This temporal effect could influence whether one particular transition to a new pattern is more difficult than another. Whilst the static rhythm is presented mechanically in the App, the player may reproduce their pattern with an element of expressivity, based on their temporal perception of the beat. However this will reduce their accuracy as assessed by the scoring algorithm. In the App, accuracy is prized over expressivity of performance.

**Fig 5:**
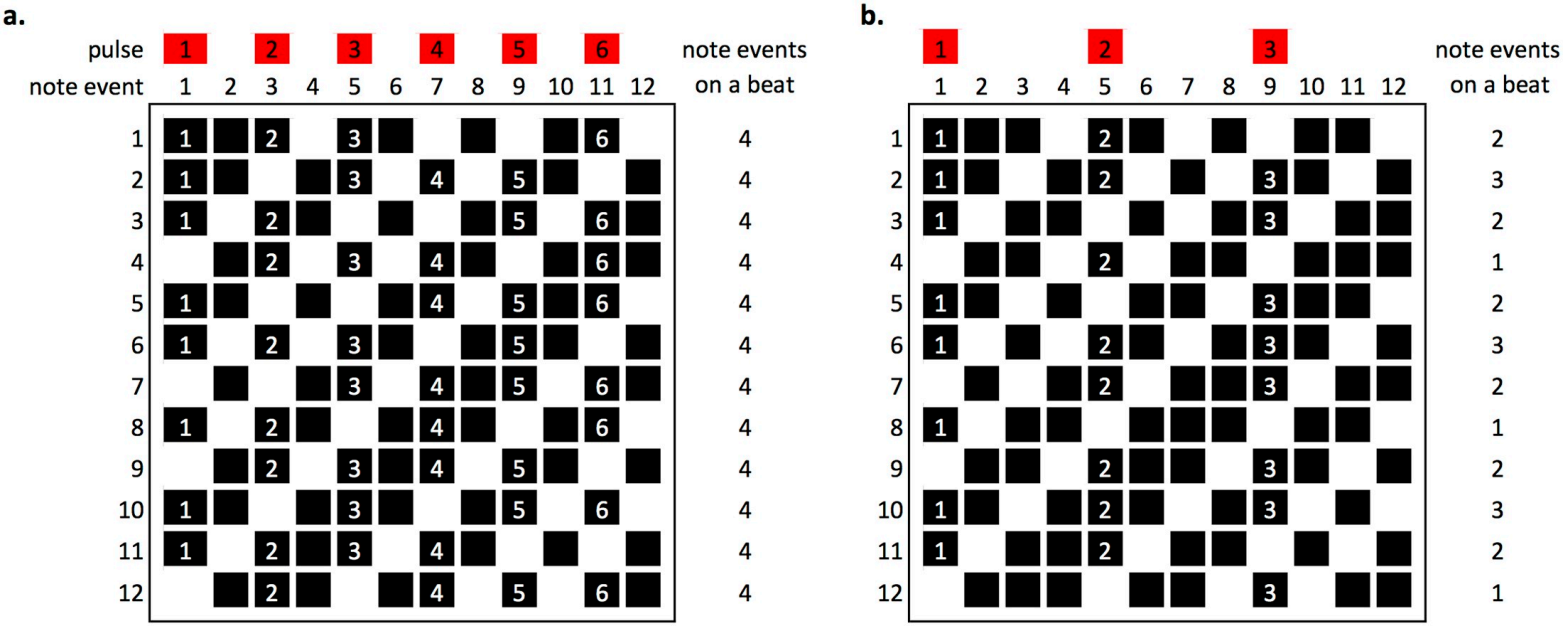
Comparing a 6/4 (a) and 3/2 (b) pulse against each of the 12 Clapping Music patterns.

At the medium and hard levels, the pulse must be determined from the static pattern alone, as in a performance. However, feedback from early game prototypes suggested that some players found this too difficult. As a result, the easy level and the training area include a metronome—representing the accented mechanical scenario described by Drake et al. [19]. However no time signature is indicated in either Reich’s original handwritten score or the formal published version (9), and the patterns display metrical ambiguity [25]. So where would an accent be helpful for a musically inexperienced new player? For example, it could be every two note events (i.e. 6 beats per bar) denoting a 6/4 time signature, every four note events denoting 3/2 or every three note events denoting 12/8 (a triplet feel). Only one metronome rule could be implemented in the game. If the metronome reinforced individual perception of where accents might be, it may make some patterns easier to reproduce, whilst having an adverse effect on patterns where the metronome fits internal beat perception less well [23]. Using a variation of box notation, developed by Philip Harland [26], it can be seen that implementing the metronome differently could influence beat perception (Fig 5). With 6 beats per pattern (6/4) each of the 12 patterns has 4 note events accented by the metronome. With 3 beats per pattern (3/2), the number of note events accented varies between 1 and 3. The 6/4 metronome was implemented as it is more likely to provide consistent influence across the different patterns.

### Pattern complexity in Clapping Music

**Fig 6:**
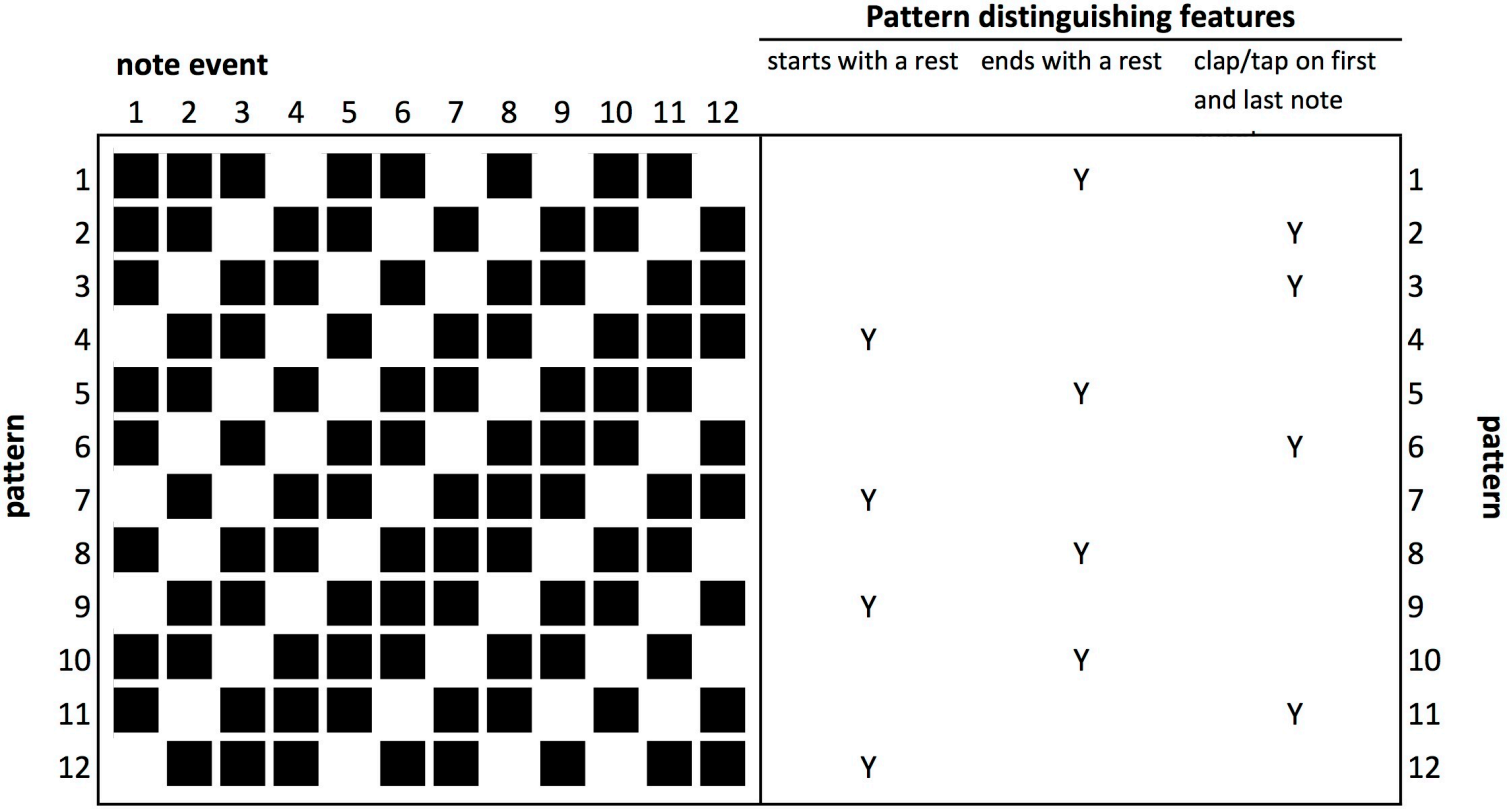
Differentiating structural features of the individual Clapping Music patterns.

We consider, in turn, two ways of assessing pattern complexity: first, using properties of the individual patterns themselves; second, using measures of the complexity of the relationship between the phasing pattern and the static pattern. First, regarding the complexity of the individual patterns, we start with the finding that it is possible to perceive a beat from temporal cues alone [18, 23]. In a study examining purely temporal effects [2, 3], reproduction performance of a rhythm was improved when regular perceptual accents were present. Metrical rhythms were easier to reproduce than non-metrical rhythms. Complex metrical patterns were harder to reproduce than simple metrical patterns (containing intervals arranged to induce a perceptual accent at the beginning of each group of four units). Applying this to Clapping Music, reproduction by players will depend on whether each pattern is defined as metrical and whether it is simple or complex. Metrical rhythms have integer ratios and the Clapping Music static pattern can be described with the integer ratios 1-1-2-1-2-2-1-2. Since each pattern is a phased variation of this rhythm, all of the patterns form integer ratios. However, not all of the rhythms have the same complexity in terms of inducing a perceived beat or accent. For example, a pattern starting with a rest is missing a key metrical accent at the beginning of the measure. Three differentiating rhythmic structures were identified: a pattern that starts with a rest on the first note event, a pattern that ends with a rest on last note event and patterns with a clap/tap on note events 1 and 12. Each pattern had one of these features (Fig 6). Through analysis of empirical data from the App we aim to determine which of these, if any, has a significant effect on ability to reproduce the patterns during the game.

**Fig 7:**
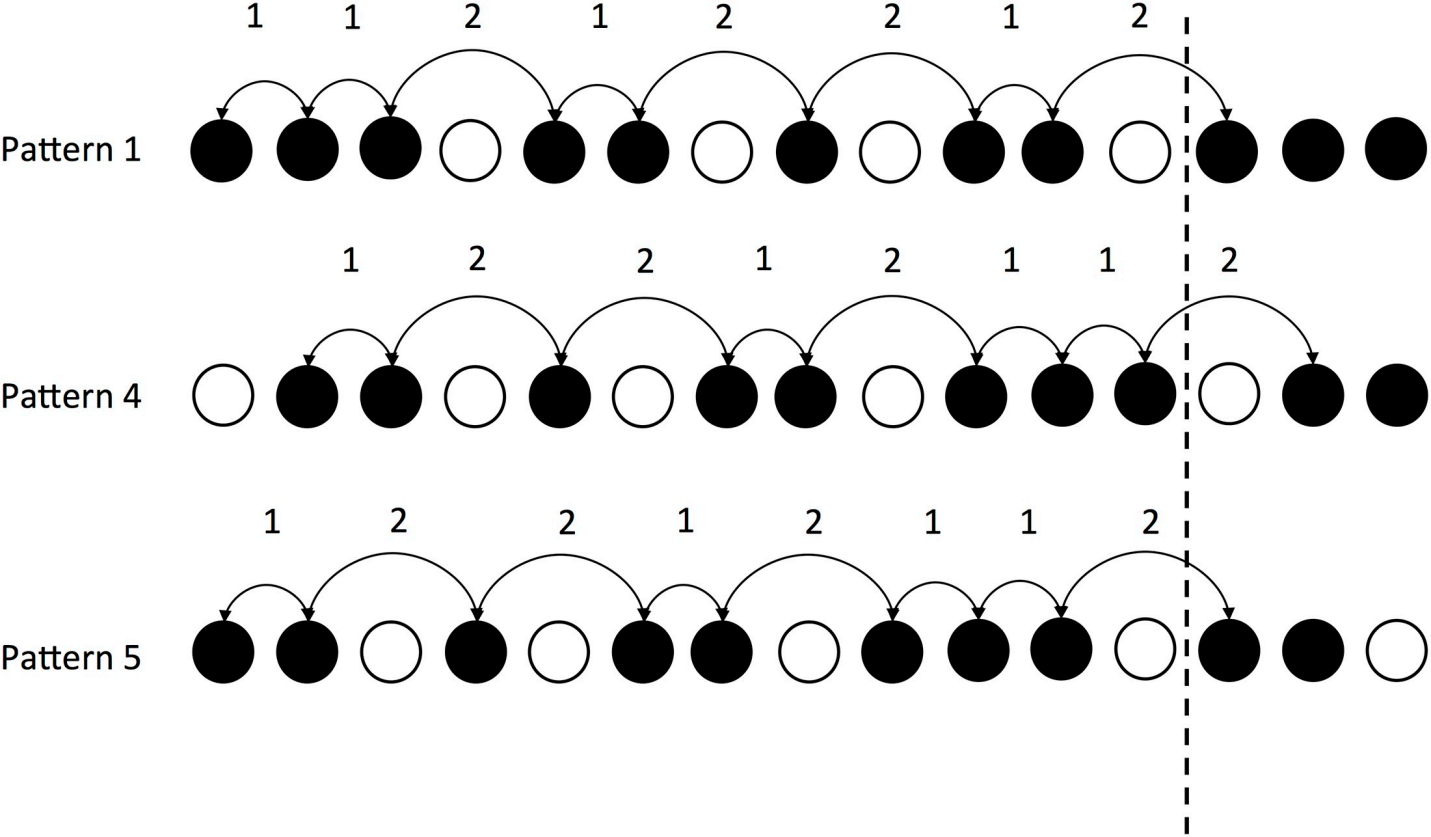
The inter-onset intervals (IOI) for the patterns in Steve Reich’s Clapping Music. The dotted line indicates the end of the first statement of the pattern and the beginning of the next repetition.

Another factor which might influence how accurately each pattern is tapped is overall rhythmic complexity. Two measures which have been adapted from the study of speech rhythms for application to music: first, the the standard deviation of the IOIs in a rhythm; and second, the normalized Pairwise Variability Index (nPVI) [27]. nPVI essentially measures the degree of contrast between successive elements in a sequence, in this case note events in a rhythmic pattern. The durational difference between adjacent intervals is measured relative to the average duration of the pair. Using this method to compare rhythms, a higher nPVI indicates greater temporal contrast between the note events in the pattern [28, ch. 3, Rhythm]. nPVI has been used for stylistic comparisons between individual composers and groups of composers defined by language, nationality and historical period [29–35]. In the case of Clapping Music, the static pattern (pattern 1) has the following sequence of IOIs: 1-1-2-1-2-2-1-2 (Fig 7). Note that since the patterns are repeated, and the ability to perform several repetitions of each pattern is of interest to us, we determine IOIs for the full pattern, to the first onset in the next iteration.

The calculation of IOI depends on being able to measure intervals from the first onset but a musical pattern may not start with an onset on the first beat. In the case of Clapping Music, four of the patterns start with a rest (Fig 6). One solution is to commence calculating intervals from the first onset that occurs for those patterns starting with a rest, as shown for pattern 4 in Fig 7. However in the case of Clapping Music, each of the twelve patterns are defined by starting on successive note events of the static pattern. Hence the interval sequence for pattern 4 (1-2-2-1-2-1-1-2) results in the same IOI sequence as for pattern 5 (1-2-2-1-2-1-1-2). This is problematic due to our hypothesis that a rest at the beginning of the pattern could make it harder to reproduce, and so is a significant differentiating factor. Examining the eight patterns that start with an onset, there is only limited variation in their nPVI, patterns 2 and 8 having a slightly higher nPVI values than the other patterns (Table 1). Examining the standard deviation of IOIs for these patterns does not distinguish between them either, every phased variation of the pattern has the same standard deviation of 0.5.

**Table 1:** Standard deviation and nPVI of IOIs for Clapping Music patterns starting with an onset.

Pattern	SD	nPVI
Pattern 1 (static)	0.5	47.6
Pattern 2	0.5	57.1
Pattern 3	0.5	47.6
Pattern 5	0.5	47.6
Pattern 6	0.5	47.6
Pattern 8	0.5	57.1
Pattern 10	0.5	47.6
Pattern 11	0.5	47.6

We turn next to measures of the complexity of the relationship between each pattern and the static pattern. One way to represent a rhythm which occurs in a stable, recurring pattern is to map it onto a circle consisting of the total number of elements or time points involved [1, 36]. This allows geometric aspects of the metre to be described. This method has been used to assess the rhythmic complexity of African, Brazilian and Cuban Clave rhythms [26], which has some relevance to Clapping Music as Steve Reich studied drumming in Ghana in 1970, prior to composing the piece in 1972, and took a particular interest in the relationship between the superimposed bell and drum patterns which underpin Ghanaian drumming [10]. As specific sources of motivation, the static pattern in Clapping Music has been related to the Yoruba bell pattern from Nigeria [25] while the fifth rotated pattern has been related to the Agbekor bell pattern of the Ewe people from Benin, Togo and southeastern Ghana [37]. Convex polygons were inscribed in an imaginary circle to geometrically analyse each rhythm, making aspects of pattern symmetry, internal right angles and reversibility accessible.

**Fig 8:**
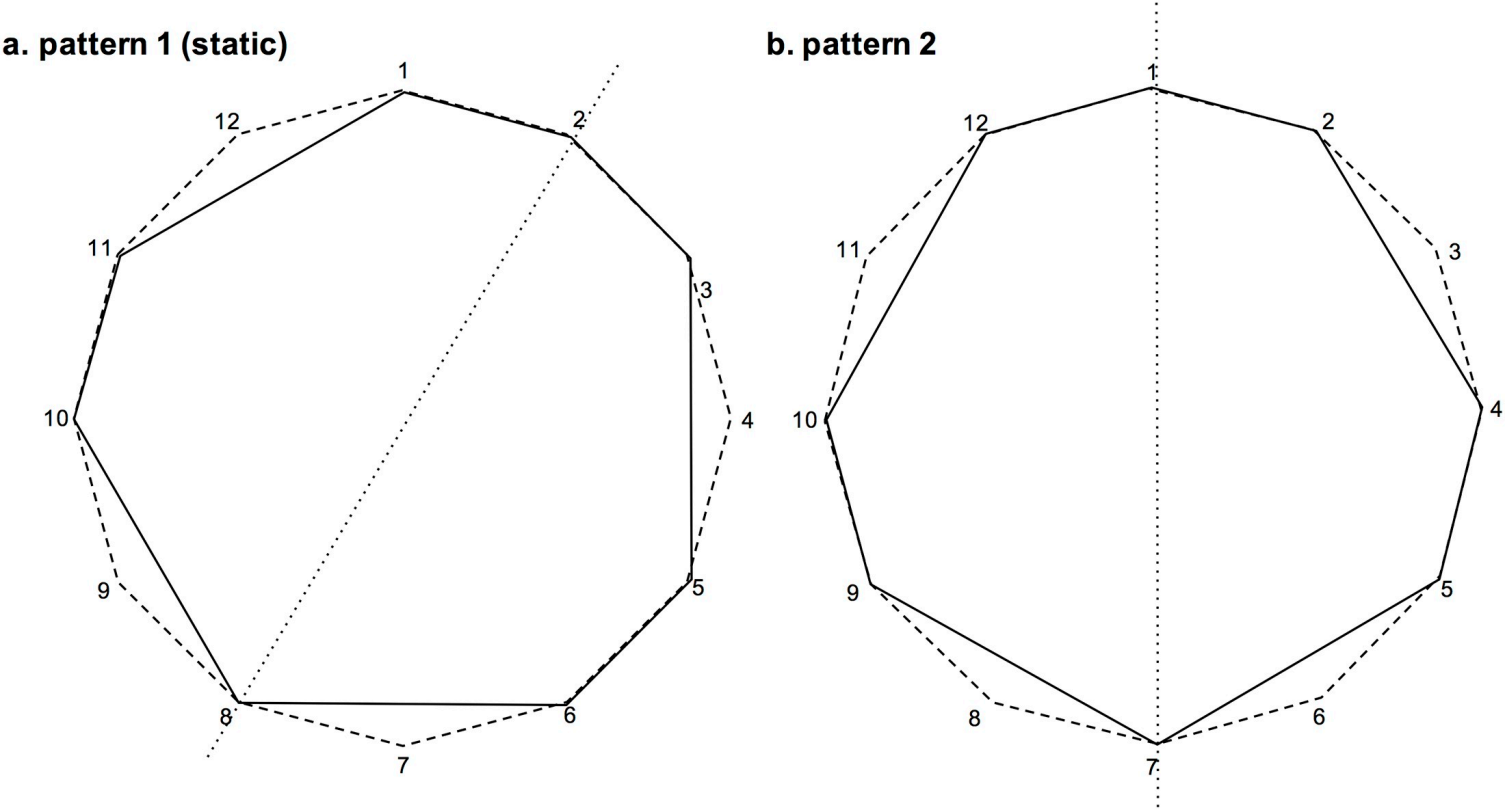
Pattern geometry described by Toussaint (2002) applied to the Clapping Music static pattern. Pattern 1—a weak palindrome, pattern 2—a palindrome.

Each of the patterns analysed by Toussaint had five note events in a 16-quaver bar (which London would describe as a 16-cycle). The static pattern in Clapping Music uses a 12-quaver bar (12-cycle) with eight note events, which has a higher note density than the rhythms considered by Toussaint, but it can still be represented in a similar way. The higher note density in the Clapping Music pattern means that all internal angles are greater than 90°, so there are no interior right angles (as were found for the African rhythms Shiko, Soukous and Gahu) however mirror symmetry could be seen about a line between notes 2 and 8 (Fig 8a). Toussaint describes this as a weak palindrome, which is also found in the Clave Son rhythm (weak since the symmetry is not around the first note of the pattern). Since the 12 patterns of Clapping Music consist of the same rhythmic cell, phased by the movement of one quaver, they all have the same geometrical shape, simply rotated within the 12-note frame. So pattern 2 has the same shape as pattern 1 but the mirror symmetry now runs through the first note (Fig 8b), which would be described by Toussaint as a palindrome (rather than a weak palindrome)—a pattern which sounds the same played backwards or forwards, as was found for Shiko and Bossa-Nova rhythms.

Toussaint goes on to use this geometric construct to analyse each of the bell and clave rhythms by comparing their internal vector distance. Counting the number of vertices skipped (i.e. rests) whilst moving round the polygon in a clockwise manner from the first onset, transforms the five note events into a five dimensional vector X = (*x*_1_,*x*_2_,*x*_3_,*x*_4_,*x*_5_). Vectors for each of the rhythms can then be compared by calculating the Euclidean Distance between them. This can be done for eight of the patterns in Clapping Music, but for the 4 patterns which start with a rest this is not possible. The calculation relies on the vector starting at a point in space, not somewhere between two points. For the 8 patterns where the Euclidean Distance can be calculated, the distance of each from the other seven patterns is identical (∑ = 14.56 Table 2). The difference between one pattern, and the pattern that it follows in the performance is also identical (∑ = 2.45 Table 2). This is not surprising since each pattern is essentially the same geometric shape, simply rotated by one note event. However since the patterns are played in ensemble and we wish to assess the difficulty of performing the phasing pattern, our interest is in each pattern’s distance from the static pattern. Patterns 5 and 8 are shown to be closest to the static pattern (1.41) whilst pattern 6 is the furthest away (2.83).

**Table 2:** The distance matrix of interval vectors with the Euclidean metric. The bottom row indicates the sum of the distance for each rhythm from the other seven.

**Pattern**	**1**	**2**	**3**	**5**	**6**	**8**	**10**	**11**
**1**	0	2.45	2.00	1.41	2.83	1.41	2.00	2.45
**2**		0	2.45	2.00	1.41	2.83	1.41	2.00
**3**			0	2.45	2.00	1.41	2.83	1.41
**5**				0	2.45	2.00	1.41	2.83
**6**					0	2.45	2.00	1.41
**8**						0	2.45	2.00
**10**							0	2.45
**11**								0
**∑**		14.56	14.56	14.56	14.56	14.56	14.56	14.56

**Fig 9:**
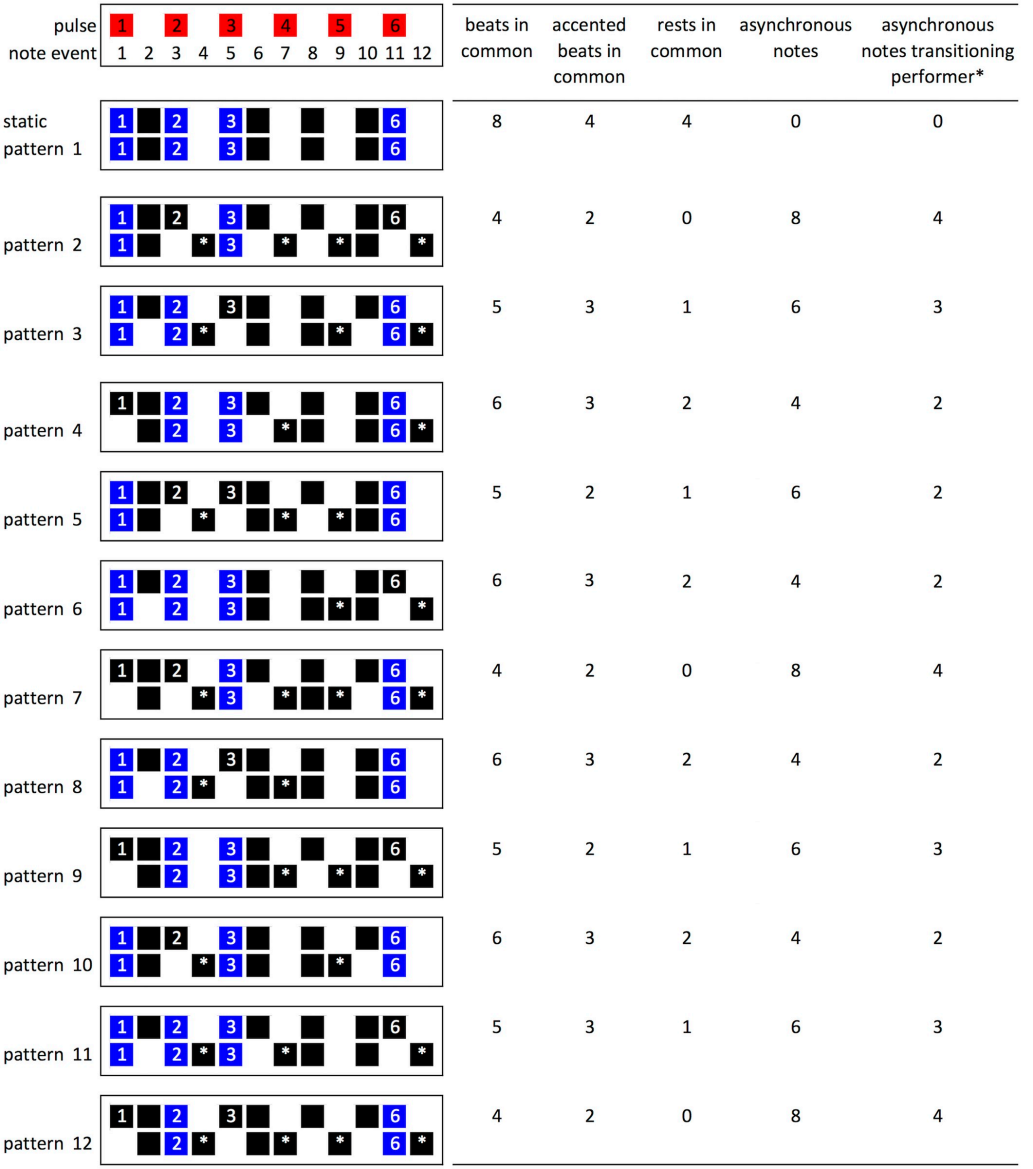
Reinforced accents resulting from the combination of static and phasing pattern according to different measures of rhythmic complexity proposed in the literature. See text for details of individual measures.

Another factor that potentially influences the difficulty of performing Clapping Music is that the pattern is not reproduced in isolation, players must be able to perceive the beat of the static pattern and produce an accurate rhythm in ensemble with it. There are only two kinds of audible events in Clapping Music: single (asynchronous) claps and double (synchronous) claps. While logically the doubles are just a sum of two singles, perceptually they are different [38]. Even if the claps are performed at exactly the same time, with as similar a timbre and volume as possible, then a double clap will differ in perceived loudness from a single clap both for listeners and performers. As a result, different pattern combinations may have different levels of complexity depending on the number of beats they have in common, rests they have in common or whether an accent is reinforced by occurring in both the static and the phasing pattern. Four patterns (4, 6, 8 and 10) have the highest number of beats in common (six beats), rests in common (2 beats) and accented beats in common (three beats) (see Fig 9).

Based on these considerations, Sethares & Toussaint [38] propose a predictor of rhythm complexity. They calculate a measure for each of the twelve patterns *c*_*i*_, which estimates the complexity of performance of each pattern (Eq 1) based on the number of single claps *s*_*i*_ double claps *d*_*i*_ and rests *r*_*i*_ (where neither performer claps):

$$\begin{array}{c} c_{i}=12-d_{i}=s_{i}+r_{i} \end{array}$$ (1)

This represents the number of time slots where doubles do not occur. For example patterns 2, 7 and 12 have the fewest double claps (4 beats in common, see Fig 9) and have *c*_*i*_ = 12 − 4 = 8 (Table 3).

**Table 3:** Predictor of rhythm complexity *c*_*i*_ applied to the twelve Clapping Music patterns [38].

Pattern	*c*_*i*_
Pattern 1	4
Pattern 2	8
Pattern 3	7
Pattern 4	6
Pattern 5	7
Pattern 6	6
Pattern 7	8
Pattern 8	6
Pattern 9	7
Pattern 10	6
Pattern 11	7
Pattern 12	8

### Implications for pattern tapping accuracy in the App

In summary there are three aspects of rhythmic complexity highlighted by Clapping Music: the first relates specifically to the complexity of the patterns themselves; the second relates to the notion of ensemble rhythm, being able to reproduce the phased patterns in time with the static pattern; and the third relates to making the transition from one pattern to the next, whilst maintaining ensemble with the static pattern. Summarising the literature reviewed above, the patterns with the greatest number of indicators of pattern complexity are hypothesised to be those that players in the game will find most challenging to reproduce; these are patterns 2, 7 and 12 (Table 4). We note that some of the methods rely on an onset on the first note event (nPVI and geometric interval vector distances) and so are less meaningful when applied to the four patterns that start with a rest (patterns 4,7,9 and 12). However, the indicators in Table 4 only reflect the first two aspects of rhythmic complexity identified; they are less helpful in determining which transition will be most difficult to complete and in fact there is scant evidence available in the empirical literature. We hypothesise that the differentiating structural characteristics of each of the 12 patterns (shown in Fig 6) will have a bearing on transition difficulty, since this determines the complexity of the transition environment. We will use performance accuracy data collected from players of Steve Reich’s Clapping Music App to investigate these hypotheses.

**Table 4:** Factors indicating pattern complexity from the literature. P indicates pattern factors, E indicates ensemble factors.

		Pattern	1	2	3	4	5	6	7	8	9	10	11	12
P	Fig 6	Starts with a rest				x			x		x			x
P	Table 1	High nPVI		x						x				
E	Fig 9	Least number of beats in common		x					x					x
E	Fig 9	Least number accented beats in common		x			x		x		x			x
E	Fig 9	Most asynchronous notes for transitioning performer		x					x					x
E	Table 2	Greatest geometric distance from static		x				x					x	
E	Table 3	Highest value of *c*_*i*_		x					x					x

## Method

Data collection was managed using Parse, an open source platform for collecting data from smart device applications. Whenever a player’s device (iPad, iPhone or iPod Touch) was connected to the Internet, their game history including tapping accuracy, playing duration and frequency, use of the training area and progress in the game was sent to the remote Parse server. If they were not connected to the Internet when the game finished, the data was retained and uploaded once the device was online again.

Users were free to choose their own player nickname. These were not verified centrally so duplicates were possible, for example we had over 80 separate players who had chosen the nickname ‘Josh’. However, it was important that there was a way to identify the gameplay history for each individual player, by aggregating separate sessions of play over time. We also needed to be able to link gameplay history to online questionnaires, which were collected separately outside the App. A unique 36 character anonymous reference code was generated by the app combining details of the user and their device. It was not visible to players and did not contain any identifying information (see [11] for more details). However, when players clicked through to the research survey from a pop-up in the app, their submission included their unique gameplay code. The survey data are not analysed here but will be investigated in future research (see [11] for preliminary analysis). The data analysed in the present research consists of over 46 million rows of gameplay data collected between 9th July 2015 and 19th July 2016, during which time 109,303 players downloaded the app and created a nickname.

Before data collection commenced, prior ethical approval was obtained from the Research Ethics Committee at Queen Mary University of London (QMREC1515a, QMERC2015/42). It was made explicit to users both in the App and on the research hub, that this data would be collected (see http://cogsci.eecs.qmul.ac.uk/clappingmusicresearch/). All data collected were downloaded and stored securely on servers at Queen Mary University of London. Data analysis was performed using the open source, statistical software, R. The individuals shown in Fig 2 have given written informed consent (as outlined in PLOS consent form) to publish the image and caption.

## Results

### The dataset

2,620,303 games were played, including sessions in the training area (Table 5). Whilst game duration in seconds was available, a more useful indicator of game length is the number of pattern rows (or *loops*) played, as loops are not sensitive to the varying tempo at each *difficulty level* (see Table 6). The total number of loops, or *loopcount*, can be calculated per game or per player nickname. The minimum loopcount required to complete the game, if all taps are consistently of a high accuracy, is 80. However a player tapping with a low or inconsistent accuracy will require more loops, as they will not be offered transitions to the next pattern until an accuracy threshold has been maintained for a period of time.

**Table 5:** Gameover flag indicating how each game ended.

gameover flag	game count	% total games	description
0	228,640	9%	games exited early
0	378,540	14%	sessions played in the training area
1	1,928,641	74%	game ended normally but incomplete
2	84,482	3%	completed games
**total**	**2,620,303**		

**Table 6:** Levels of difficulty.

difficulty flag	level	proportion of games played	tempo (bpm)	average accuracy
-1	practice game	14%	variable	0.54
0	game at easy level	44%	130	0.60
1	game at medium level	12%	140	0.75
2	game at hard level	30%	155	0.84

The *gameover* variable indicates how each game ended: 0 = a practice session or a game that was exited before it ended (e.g. because the player chose to stop playing mid-game, or the app crashed); 1 = game ended before pattern 13 as player accuracy was not maintained; 2 = game completed (i.e. player successfully completed patterns 1 to 13). The majority of games (74% Table 5) ended normally but incomplete (i.e. the game ended before the player could finish all 13 patterns). Examining the maximum value of gameover for each player indicates that 19% of players completed a game at least once, at one of the difficulty levels, and 5% of players completed the game at the hardest level (difficulty level 2, see Table 6). The data for each game included a flag indicating the level of difficulty chosen by the player. The largest number of games were played at the easy level, the medium level being played the least. Tempo and the tapping accuracy threshold in the scoring algorithm were set higher for each level of difficulty. This was reflected in the average gameplay accuracy achieved for each level (Table 6).

### Evidence of motivation to complete the game

The majority of games were completed at the easy level (Table 7). 66% of players were motivated to continue playing after completing the game for the first time (Table 8). 54% of players completed games at just one level of difficulty (easy 40% medium 7% hard 7% Table 9). However many players completed games at more than one level, 14% completed at least one game at each of the three levels of difficulty (Table 9). Whilst the smallest number of players completed a hard game (5,685 players, Table 7), they completed more games per person than at any other level of difficulty (5 games per player, Table 7). Even having completed the game at the hardest level, some players were then motivated to keep playing to increase their final score [11, pp. 39-44].

**Table 7:** Number of games completed at each level of difficulty. Note that some players played the game at more than one level of difficulty, hence there are fewer unique players in total than the sum of players at each level.

difficulty level	number of games completed	proportion of completed games	number of players at each level	average number of completed games per player
easy	34,987	42%	16,981	2
medium	21,297	25%	10,157	2
hard	28,198	33%	5,685	5
**total**	**84,482**	**100%**	**20,581**	**4**

**Table 8:** Number of games completed by individual players.

	number of players	%
completed 1 game only	6,986	34%
completed 2-5 games	9,828	47%
completed 6-10 games	2,389	12%
completed 11-20 games	965	5%
completed 21 or more games	413	2%
completed 50 or more games	67	0.03%
**total players**	**20,581**	

**Table 9:** Number of players who completed games at more than one level.

Levels completed	number of players	%
Easy only	8,218	40%
Easy and Medium	5,274	26%
Easy, medium and hard	2,791	14%
Hard only	1,508	7%
Medium only	1,404	7%
Easy and Hard	698	3%
Medium and Hard	688	3%
**total players**	**20,581**	

### Assessing pattern difficulty using average tap accuracy

The average tap accuracy of all loops played for each pattern was analysed to investigate whether any patterns in particular appeared to be more difficult for players. To recap, tap accuracy is measured on a scale of 0.00 to 1.00, where 1.00 indicates that the player has tapped one loop of the expected pattern with perfect timing, and no omitted or additional taps with reference to the expected pattern. It was noted that accuracy was significantly lower for patterns 1 and 2 than for any other pattern (Table 10).

**Table 10:** Average accuracy, proportion of loops played and the proportion of games which ended on each pattern, for all games, all levels of difficulty.

pattern	accuracy	proportion of loops played	proportion of games ended incomplete
1	0.66	47%	62.1%
2	0.68	14%	21.6%
3	0.79	8%	4.1%
4	0.80	6%	3.0%
5	0.80	5%	1.8%
6	0.81	4%	2.2%
7	0.83	4%	1.5%
8	0.79	3%	1.1%
9	0.80	2%	1.2%
10	0.82	2%	0.5%
11	0.83	2%	0.5%
12	0.81	2%	0.4%
13	0.83	1%	0.1%

Players must methodically progress through the patterns in the same order as indicated in the score, always starting with pattern 1. During the first pattern players are getting settled into the game and it is difficult to start with high accuracy from the very first tap. Pattern 1 represented 47% of all loops played. This is partly because Pattern 1 requires a minimum of 8 repetitions whereas the other patterns require a minimum of just 6. However this is also because for many of the games, the player did not progress beyond pattern 1 (62% of all games ended on pattern 1, Table 10). There is another opportunity to assess the first pattern (which is also the static pattern) since players must repeat it when they have completed all 12 patterns in order to finish the game—patterns 1 and 13 are the same pattern. However only those players with enough skill to progress substantially in the game will get the opportunity to try pattern 13. Pattern 2 follows a player’s first transition to a new pattern, which also led to the game ending for many players (22% of games ended on pattern 2, Table 10). For some players this was part of the learning curve and as they continued playing more games they were able to progress further and eventually complete all 13 patterns. However some players never progressed beyond the first few patterns and many never got as far as pattern 13 at any level of difficulty.

The factors affecting patterns 1 and 2 highlight a problem associated with looking at the whole dataset—the difference between complete and incomplete games. A solution is to look purely at the 3% of games (Table 5) completed successfully (gameover = 2). The resulting dataset prioritises those players with enough skill to complete the game at least once (19% of players), however this ensures that all patterns were played equally for every game analysed. This removes any bias due to earlier patterns including games that ended due to low accuracy, and later patterns only being played by the more skilled players. Whilst this is only a small percentage of the total gameplay data available, the total size of the dataset means that this still represents 84,482 games (Table 5) and 7,646,176 loops played.

**Fig 10:**
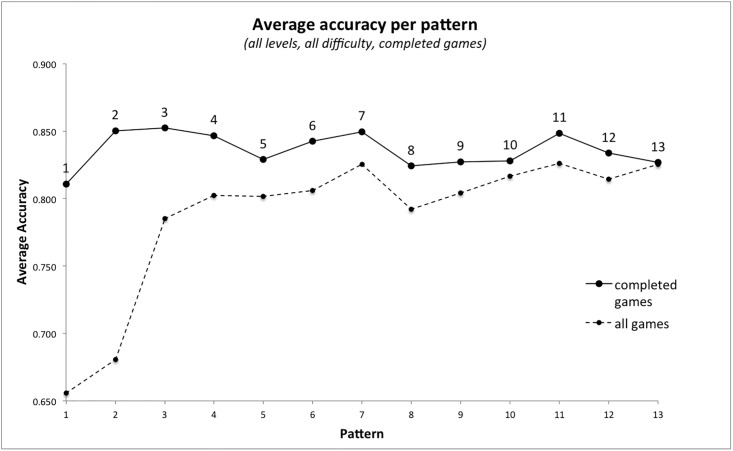
Average tap accuracy per pattern for all games, compared to completed games.

A subset of the gameplay data was created that only included completed games. Pattern 1 still had the highest proportion of loops (12% Table 11), however the proportion of loops per pattern was now much more evenly distributed overall. This had a corresponding effect on average tap accuracy per pattern (Fig 10). The accuracy for pattern 1 for completed games was 0.81 (Table 11) compared to 0.66 for all games (Table 10). The accuracy for pattern 2 for completed games was 0.85 (Table 11) compared to 0.68 for all games (Table 10). Pattern 13 average accuracy remained at 0.83 as players that got this far in incomplete games had a similar level of skill to those who managed to complete the game (they were often the same players). The analysis that follows will focus on completed games only.

**Table 11:** Average accuracy and proportion of loops played per pattern, for completed games, for all levels of difficulty.

pattern	accuracy	average loops played per pattern	proportion of loops played
1	0.81	11	12%
2	0.85	7	7%
3	0.85	6	7%
4	0.85	7	7%
5	0.83	7	7%
6	0.84	7	7%
7	0.85	7	8%
8	0.82	7	7%
9	0.83	7	8%
10	0.83	7	7%
11	0.85	7	7%
12	0.83	7	7%
13	0.83	6	7%

### Does tap accuracy differ between patterns?

**Fig 11:**
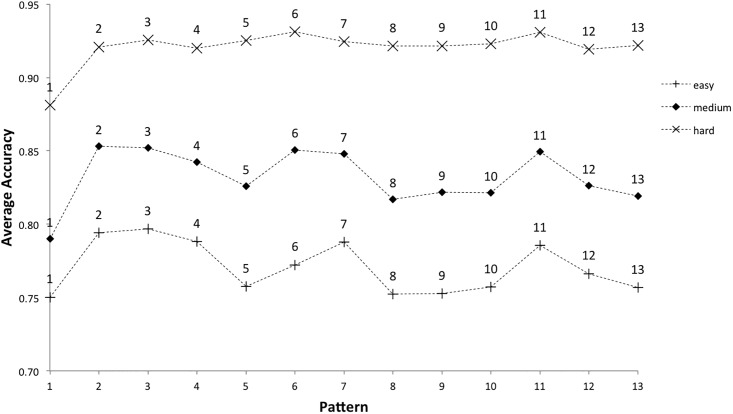
Average accuracy per pattern, by difficulty level, for completed games (gameover = 2). Note that a line is drawn between the values of average accuracy per pattern, highlighting the gameplay profile for each level of difficulty. Whilst these are discrete ordinal data points rather than continuous data, and would not usually be linked in this way, it is helpful for purposes of visual illustration.

After pattern 1, the patterns with the lowest average accuracy for completed games were patterns 5, 8, 9, 10, 12 and 13 (Table 11 and Fig 10). Of these, only pattern 12 had a high number of indicators of pattern complexity from the literature (Table 4). Average accuracy for completed games was submitted to a two-way ANOVA with the independent variables *difficulty level* (three levels: easy, medium and hard) and *pattern number* (1-13). Tap accuracy was significantly influenced by pattern number, F(1,12) = 2,834, p < .01, and level of difficulty, F(1,2) = 392,840, p < .01, with a significant interaction also evident, F(1,24) = 465, p < .01. Looking at the difference between tap accuracy per pattern at each level of difficulty, patterns 5, 8, 9, 10, 12 and 13 were the least accurately tapped at the easy and medium level of difficulty, but there were some differences at the hardest level (Fig 11).

The variability in accuracy score is low at the hardest level: after pattern 1, average tap accuracy falls in a range of just two data points 0.92-0.93. Tap accuracy for the easy and medium levels, after pattern 1, fall in a wider range (0.75-0.80 and 0.82-0.85 respectively). It seems that players completing the game at the hardest level are tapping accurately and consistently, with little distinguishing between pattern accuracy, whilst players at the easy and medium level are more sensitive to the specific pattern being played. Players at the hardest level of difficulty are no longer playing to complete the game, they are playing competitively to increase their overall score, only continuing with games that contain no error and might score 28,000 or above [11]. Next we will look in more detail at one of the specific challenges of Clapping Music which might influence pattern accuracy; managing the transition to a new pattern whilst staying in ensemble with the static pattern.

### Measuring transition difficulty

Loops played in the game are numbered sequentially each time a player starts a new pattern. For example, pattern 1 starts with loops 0,1,2,3,4,5,6,7 (minimum of 8 repetitions required), pattern 2 starts with loops 0,1,2,3,4,5 (minimum of 6 repetitions required), and so on. It is possible to investigate how problematic a transition is by examining the accuracy per loop number for the minimum number of loops that have to be played for each pattern. Average accuracy for completed games was submitted to a two-way ANOVA with the independent variables *loop number* (0-5) and *pattern number* (1-13). The results showed that loop number, F(1,5) = 28,313, p < .01, and pattern number, F(1,12) = 11,264, p < .01, had a significant impact on accuracy with a significant interaction also evident, F(1,60) = 10,292, p < .01.

**Fig 12:**
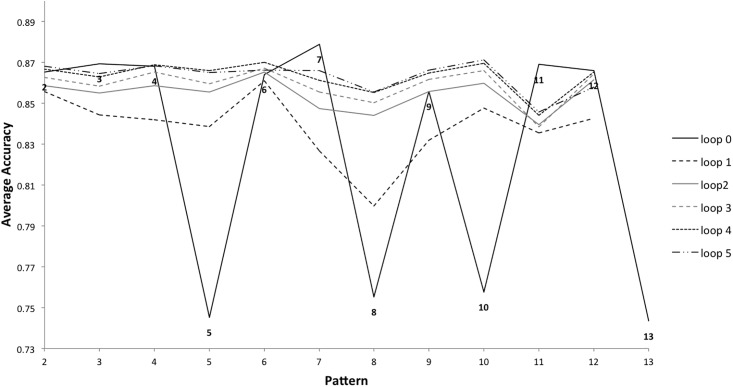
Average accuracy per loop, per pattern, for loops 0-5 of completed games. Pattern 1 is excluded.

The first loop played (loop 0) for each of the patterns showed the highest variation in average accuracy across each pattern (see Fig 12). This suggests that patterns with lower average tap accuracy for loop 0 are part of a more difficult transition, from one pattern to the next. Pattern 1 had the lowest loop 0 tap accuracy by some way (0.300). As discussed previously, it is difficult to tap with high accuracy as soon as the game starts, and loop 0 of pattern 1 is the very first loop played for every game, so this is unsurprising. For this reason, pattern 1 has been excluded from Fig 12. After this, patterns 5, 8, 10 and 13 have the lowest average accuracy for loop 0, implying these were the hardest transitions overall. The second loop (loop 1) also showed some variation in average accuracy per pattern. By the third loop (loop 2), accuracy became more consistent by pattern. This suggests that players recover from transitions quickly, within one or two loops. It was noted that loop 1 also had a unique profile for tap accuracy per pattern compared to the other loops analysed, in that pattern 9 had the lowest average tap accuracy, which had not been the case for loop 0 (Fig 12). It is not clear why this pattern should be problematic specifically for for the second loop played after the transition; it did not have reduced tap accuracy for the first loop played, or the following loops.

**Fig 13:**
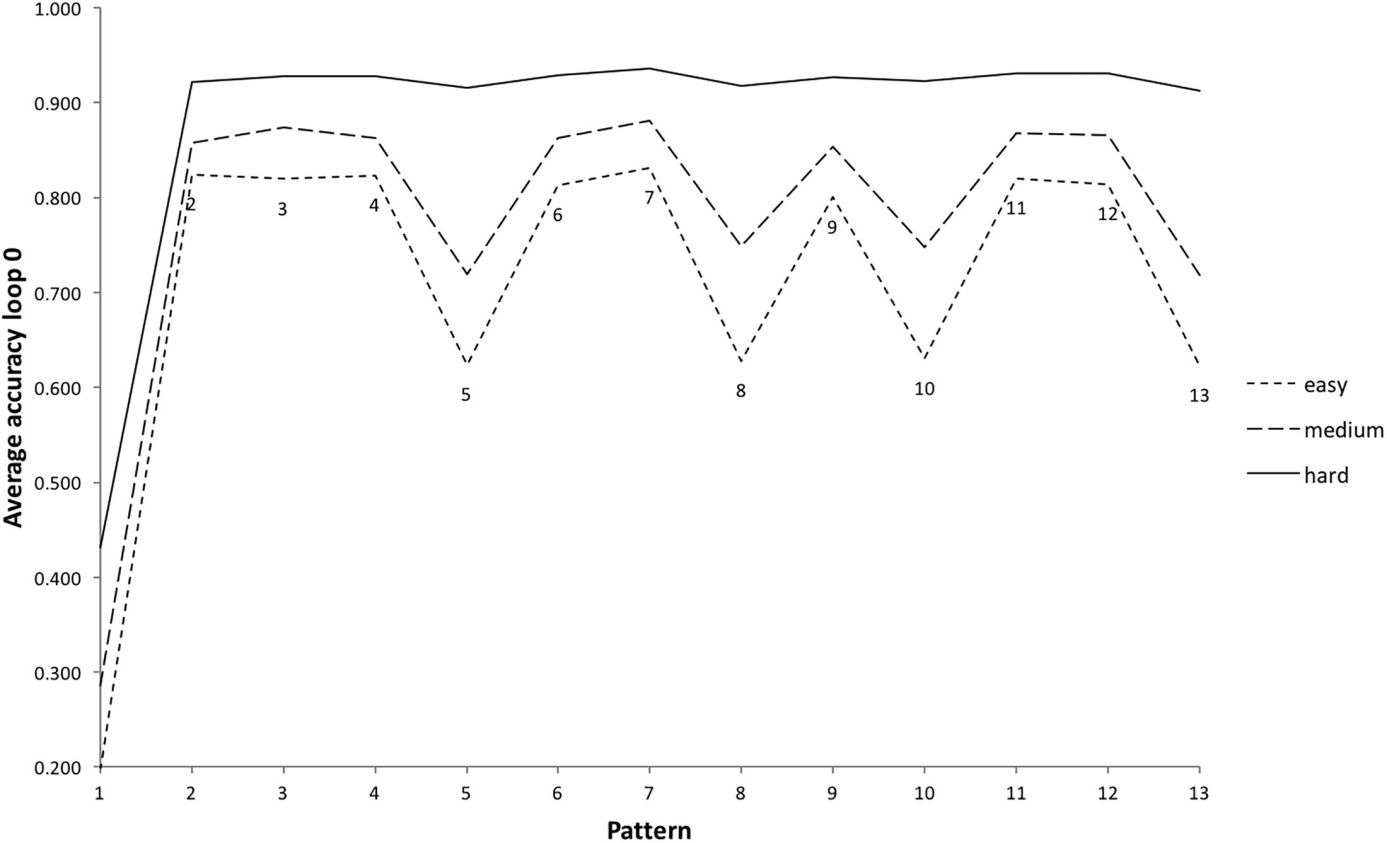
Average accuracy per pattern for first row played (loop 0), completed games (gameover = 2).

Average accuracy for loop 0 in completed games was submitted to a two-way ANOVA with the independent variables *difficulty level* (easy, medium and hard) and *pattern number* (1-13). The results showed that difficulty level, F(1,2) = 65,058, p < .01, and pattern number, F(1,12) = 43,141, p < .01, had a significant impact on accuracy for loop 0, with a significant interaction also evident, F(1,24) = 1,252, p < 0.01. The variation in accuracy per pattern for loop 0 was greater at easy and medium levels than at the hard level (Fig 13). Once again, tap accuracy for easy and medium games differentiates between the patterns, whilst at the hard level this is not so marked.

Pairwise t-tests were carried out on the average loop 0 tap accuracy for pairs of patterns, ranked by ascending tap accuracy for each level of difficulty. Pattern 1 has a significantly different loop 0 tap accuracy to the next least accurate pattern (pattern 13 in each case), at each level of difficulty (p < .05 Table 12). At the easy level of difficulty, patterns 13, 5, 8 and 10 are not significantly different from each other in terms of loop 0 tap accuracy, but are all significantly different from the next ranked pattern, 9. At the medium level of difficulty, patterns 13 and 5 are not significantly different from each other, but are significantly different from the next least accurate pattern, 10. Patterns 8 and 10 are significantly different from the next least accurate pattern, 9. At the hard level of difficulty the difference in means between the patterns is not significant, except for patterns 11 and 7. This is consistent with the reduced variability seen between patterns at the hard level (Fig 13).

**Table 12:** Average tap accuracy per pattern, by difficulty level, for loop 0 for completed games. Patterns are ranked by increasing average tap accuracy along with Bonferroni-adjusted p-values from t-tests comparing the accuracy for a pattern with the accuracy for the pattern immediately above it. Shading indicates groups of patterns whose accuracy does not differ significantly (using an alpha level of 0.05).

Easy			Medium			Hard			All levels		
patt.	average acc.	p-value	patt.	average acc.	p-value	patt.	average acc.	p-value	patt.	average acc.	p-value
1	0.20		1	0.29		1	0.43		1	0.30	
13	0.62	0.00	13	0.72	0.00	13	0.91	0.00	13	0.74	0.00
5	0.62	1.00	5	0.72	1.00	5	0.92	0.92	5	0.75	1.00
8	0.63	1.00	10	0.75	0.00	8	0.92	1.00	8	0.76	0.00
10	0.63	1.00	8	0.75	1.00	2	0.92	0.28	10	0.76	1.00
9	0.80	0.00	9	0.85	0.00	10	0.92	1.00	9	0.86	0.00
6	0.81	0.00	2	0.86	1.00	9	0.93	0.19	6	0.86	0.00
12	0.81	1.00	6	0.86	1.00	4	0.93	1.00	2	0.87	1.00
3	0.82	0.66	4	0.86	1.00	3	0.93	1.00	12	0.87	1.00
11	0.82	1.00	12	0.87	1.00	6	0.93	1.00	4	0.87	1.00
4	0.82	1.00	11	0.87	1.00	12	0.93	1.00	11	0.87	1.00
2	0.82	1.00	3	0.87	0.63	11	0.93	1.00	3	0.87	1.00
7	0.83	0.01	7	0.88	0.06	7	0.94	0.01	7	0.88	0.00

In summary, the results demonstrate that average tap accuracy is significantly impacted by the loop number and level of difficulty. Players recover quickly from transitions and loop 0 is an indicator of the transitions that were most challenging, indicating that the transitions to patterns 5, 8, 10 and 13 were the most difficult. The results also show that some patterns have significantly different loop 0 average tap accuracy compared to their closest ranked neighbours. Next we examine the structure of transitions between rhythmic patterns to understand why some transitions present more of a challenge than others.

### Understanding difficult transitions

**Fig 14:**
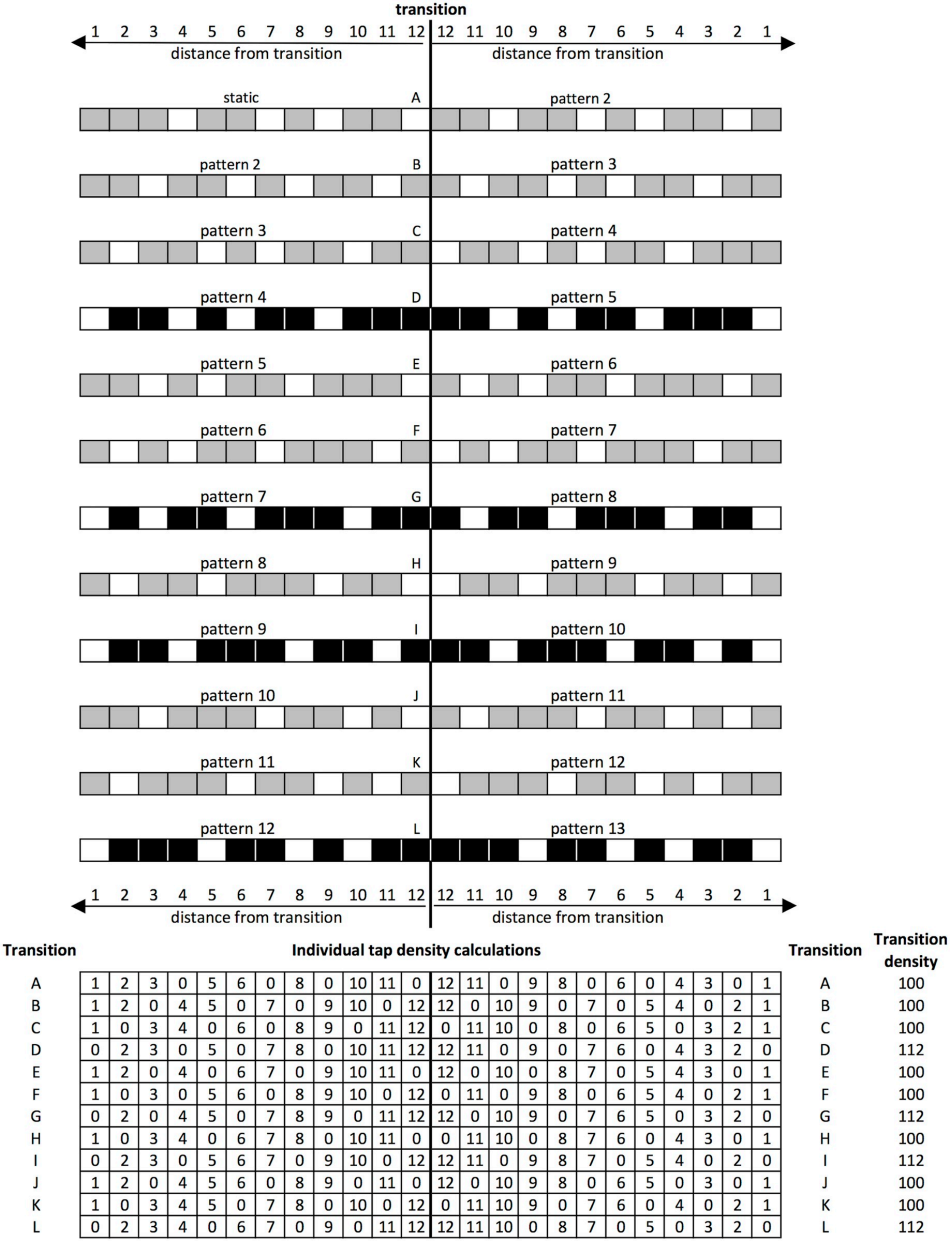
A representation of transition tap density, showing the two patterns either side of the transition boundary (marked with a vertical line). Grey cells represent taps, white cells represent rests. Transitions with the highest tap density are shown in black. Tap density is calculated for each transition by multiplying note event (tap/clap = 1, rest = 0) with distance from transition (0–12, 12 being the closest to the transition, 1 being the furthest away in either direction).

Patterns 5, 8, 10 and 13 (the static pattern repeated) had the fewest indicators of pattern complexity from the literature (Table 4), but the lowest loop 0 tap accuracy (Fig 12). The transitions into these patterns are characterised by having three or more taps in a row, spanning the transition (transitions D, G, I and L Fig 14). Therefore, we developed a quantitative measure of tap density to differentiate between these transitions. For the two patterns either side of each transition, each of the twelve possible note positions was assigned a score of ‘1’ if there was a note at that position and ‘0’ otherwise (i.e., for a rest). This was then multiplied by the position of the tap in relation to the transition boundary. In this way a tap closer to the transition had a higher tap density. The score for each note position was then totalled for each transition in order to obtain an overall score, which differentiated between transitions with respect to the number of taps closest to the transition boundary. It was found that the transitions with the highest tap density relative to the transition boundary, were the same transitions that had the lowest average accuracy for the first loop after the transition—D, G, I and L (Fig 14). In other words, the transitions that players found most difficult to recover from were also those with the greatest tap density immediately before and after the point of transition.

**Fig 15:**
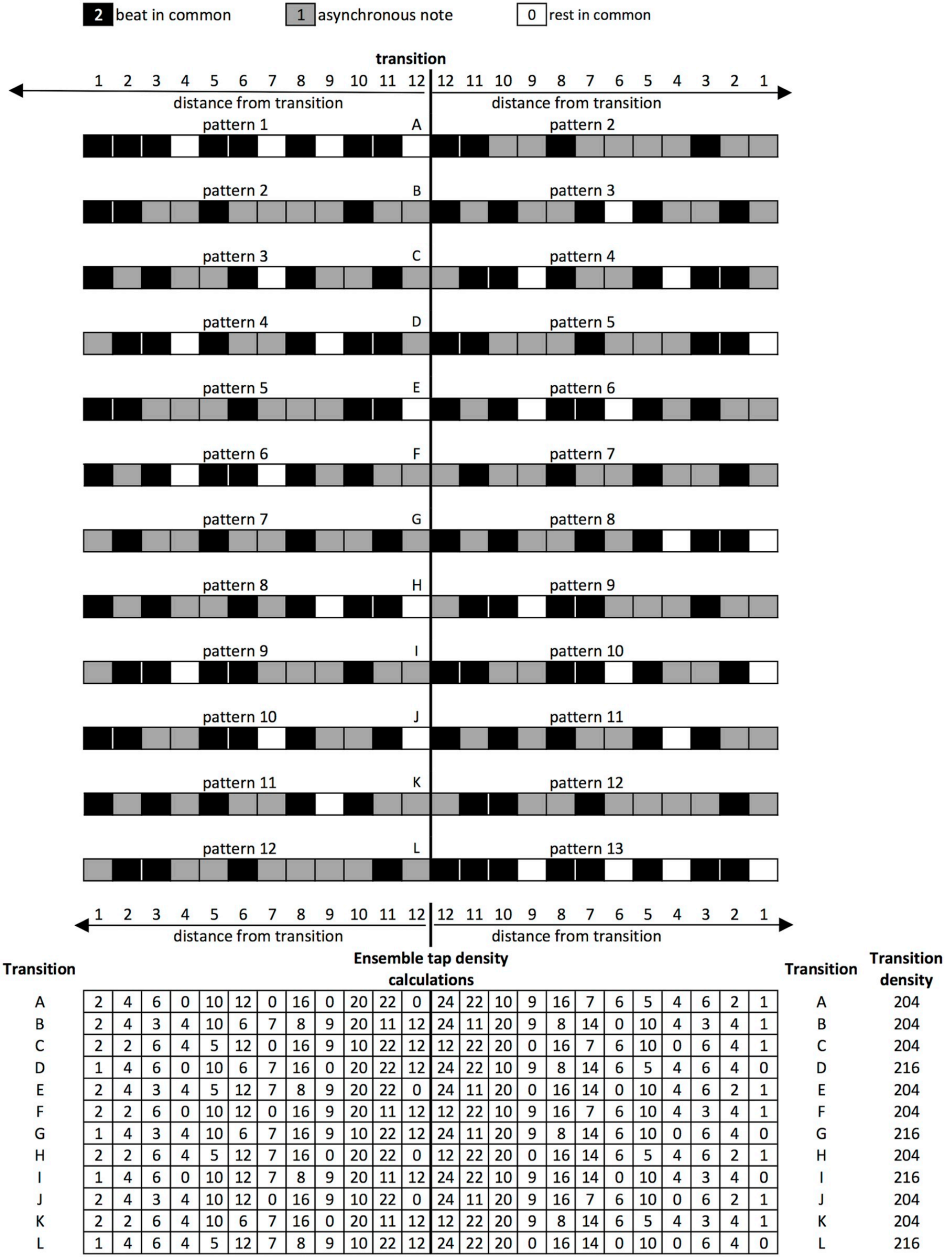
A representation of ensemble transition tap density. Calculated by multiplying each ensemble note event (beat in common = 2, asynchronous note = 1, rest in common = 0) with distance from the transition (0–12, 12 being the closest to the transition, 1 being the furthest away in either direction).

Not forgetting that transitions occur in ensemble with the static pattern, we also calculated an ensemble tap density. A beat in common had a score of 2, an asynchronous note a score of 1, a rest in common a score of 0. Again these scores were multiplied by the distance of the note event from the transition, in either direction. Transitions with the highest ensemble tap density were again the transitions with the highest pattern transition tap density and the lowest accuracy for the first loop after the transition (D, G, I and L Fig 15).

### Excluding the transition effect

We have seen that loops 0 and 1 show unique effects in terms of tap accuracy per pattern, reflecting the structural aspects of transitions presenting the greatest challenge to players. Removing these loops may tell us something about the inherent difficulty of each pattern, excluding the transition effect. This analysis is no longer limited to the minimum number of loops that must be played, loops 0-6. Now we can consider all loops played after loop 1. To recap, if a player does not meet the accuracy threshold set in the scoring algorithm for each level of difficulty, they have an opportunity to keep playing and improve. If tap accuracy continues to decline the dots move up the screen, and if they reach the top then it is game over. If tap accuracy improves the dots move back down the screen, the next transition being made available once they reach the bottom again (Fig 4). So for a pattern that the player is finding difficult, they will play more than 6 loops before regaining the accuracy required to continue. As we are only examining completed games, we know that the player did eventually regain the accuracy required in each case.

**Fig 16:**
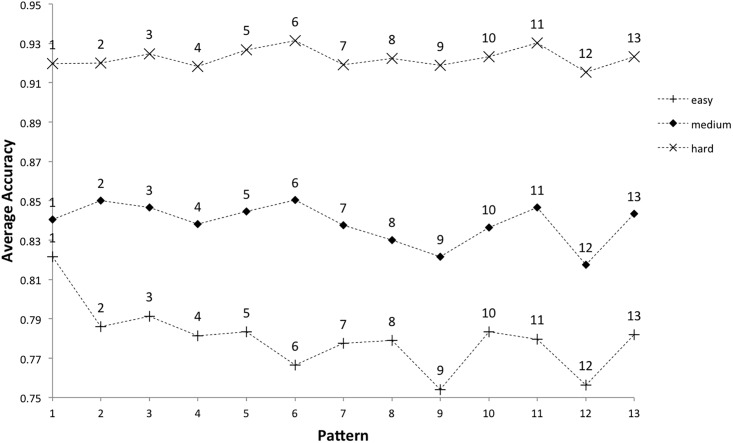
Average accuracy per pattern by level of difficulty, for completed games excluding loop 0 and loop 1.

Average accuracy per pattern excluding loops 0 and 1 for completed games was submitted to a two-way ANOVA with the independent variables difficulty level (easy, medium and hard) and pattern number (1-13). The results showed that difficulty level, F(1,2) = 291,905, p < .05, and pattern number, F(1,12) = 1,553, p < .05, had a significant impact on pattern tap accuracy with a significant interaction also evident, F(1, 24) = 568, p < .05. Where previously patterns 5, 8, 9, 10, 12 and 13 were the most difficult overall, now patterns 9 and 12 appear to have the lowest average tap accuracy (Fig 16).

Pairwise t-tests were carried out on the average tap accuracy per pattern excluding loops 0 and 1, ranked by ascending tap accuracy for each level of difficulty. Pattern 1 is no longer the least accurate pattern; at the easy level it is now the most accurate (Table 13). Patterns 9 and 12 are consistently the least accurate across each level of difficulty, with the exception of the hard level where pattern 4 is ranked above pattern 9. Again, the hard level shows the least variability. At the easy level, the mean average accuracy is not significantly different between patterns 9 and 12, however they are both significantly different to the next ranked pattern, 6. Looking at the three least accurate patterns at the medium level, mean tap accuracy is significantly different for patterns 12 and 9, and patterns 9 and 8. Overall, the mean average accuracy is not significantly different between patterns 9 and 12, however they are both significantly different to the next ranked pattern, 8.

**Table 13:** Average tap accuracy per pattern, by difficulty level, for completed games excluding loop 0 and loop 1. Patterns are ranked by increasing average tap accuracy along with Bonferroni-adjusted p-values from t-tests comparing the accuracy for a pattern with the accuracy for the pattern immediately above it. Shading indicates groups of patterns whose accuracy does not differ significantly (using an alpha level of 0.05).

Easy			Medium			Hard			All levels		
patt.	average acc.	p-value	patt.	average acc.	p-value	patt.	average acc.	p-value	patt.	average acc.	p-value
9	0.75		12	0.82		12	0.92		12	0.83	
12	0.76	0.34	9	0.82	0.00	4	0.92	0.00	9	0.83	1.00
6	0.77	0.00	8	0.83	0.00	9	0.92	1.00	8	0.84	0.00
7	0.78	0.00	10	0.84	0.00	7	0.92	1.00	6	0.84	1.00
8	0.78	1.00	7	0.84	1.00	1	0.92	1.00	7	0.84	0.00
11	0.78	1.00	4	0.84	1.00	2	0.92	1.00	10	0.84	1.00
4	0.78	1.00	1	0.84	0.06	8	0.92	0.00	4	0.84	1.00
13	0.78	1.00	13	0.84	0.00	10	0.92	0.72	13	0.84	1.00
10	0.78	1.00	5	0.84	1.00	13	0.92	1.00	11	0.84	1.00
5	0.78	1.00	11	0.85	1.00	3	0.92	0.00	5	0.84	1.00
2	0.79	0.08	3	0.85	1.00	5	0.93	0.00	2	0.85	1.00
3	0.79	0.00	2	0.85	0.00	11	0.93	0.00	3	0.85	0.00
1	0.82	0.00	6	0.85	1.00	6	0.93	0.03	1	0.87	0.00

There are more differences between difficulty levels now, for example pattern 6 is the most accurate at medium and hard levels (implying it was the easiest to tap consistently) but is the third hardest at the easy level. However, patterns 9 and 12 emerge as the least accurately tapped, and so the patterns found most difficult by players. So whilst difficult transitions reduce tap accuracy for patterns 5, 8, 10 and 13; patterns 9 and 12 appear to be the most inherently difficult.

## Discussion

Previous studies of rhythmic pattern complexity have used both mechanical [2, 3] and expressive [19, 38] audio stimuli. There has been significant mathematical analysis of Clapping Music to determine ways to differentiate between the patterns [25, 39, 40]. However to date there has been very little empirical evidence showing which patterns are found to be the most difficult to perform, or attempting to isolate the three factors involved: inherent pattern complexity, playing in ensemble with the static pattern and making the transition from one pattern to another. Moreover, most psychological studies of rhythm production more generally have focused on reproduction of artificial stimuli by small numbers of participants under laboratory conditions. By contrast, we have collected large amounts of data under ecologically valid conditions using a freely available game-based app to investigate the rhythmic properties that account for variations in performance accuracy of the different patterns in Clapping Music. In spite of the apparent simplicity of Clapping Music, it is a challenging piece to perform and the app generated a very large and complex set of data to analyse. Nonetheless, this research has delineated, and provided empirical evidence for, the factors influencing the difficulty of performing individual rhythmic patterns and transitions between patterns.

None of the identified measures of rhythmic complexity consistently accounted for differences in performance accuracy between the patterns. However, the fine granularity of the data collected, at a single loop level, allowed the transition effect to be isolated from the inherent complexity of the patterns themselves, leading to two key findings. First, that transitions to patterns 5, 8, 10 and 13, were the most challenging for players. Calculating transition tap density in relation to the transition point, for both the transitioning patterns in isolation, and in ensemble with the static pattern, suggested that this was a strong indicator of transition difficulty. Transition difficulty has not been studied in previous research and yet it was an important determiner of performance in the present study. Furthermore, the ability to transition smoothly between rhythms, sometimes with different metrical properties, is an important ability in music performance. It would be interesting in future research to examine how well the quantitative measure of transition tap density developed here generalizes to predicting performance accuracy and perceived complexity for a wider variety of rhythmic patterns.

The second finding is that when the transition effect was removed, by excluding loops 0 and 1 from the data, two of the patterns starting with a rest (9 and 12) were inherently the most difficult for players, consistently across levels in the game. Whilst pattern 4 also starts with a rest, this exhibited a weaker effect on inherent pattern accuracy, perhaps because it has more accented beats in common with the static pattern than patterns 7, 9 and 12 (Fig 9). This explanation is incomplete however, because pattern 7 shares the same indicators of pattern complexity considered from the literature with pattern 12, and has more indicators of complexity than pattern 9 (see Table 4), but was performed with significantly higher accuracy than both of those patterns (see Table 13). Due to the way the composer transformed the patterns, through discrete phasing, the patterns starting with a rest (4, 7, 9 and 12) also led into the transitions identified as the most difficult (transitions D, G, I and L to patterns 5, 8, 10 and 13) with the highest tap density around the transition point. It is noteworthy that many of the methods used to review rhythm complexity from the literature rely on the pattern being analysed starting with an onset, and so are difficult to apply to the patterns that were found to be most difficult here (i.e., those starting with a rest). This suggests the need for new ways to assess rhythmic complexity of music which take into account factors such as rhythms that start with a rest, the ensemble effect of rhythms in a musical context and the transition from one pattern to another.

A professional percussionist with significant experience of performing pieces by Steve Reich, including Clapping Music, provides some independent insight into how transitions with a high tap density are experienced from the perspective of the transitioning performer (T. Palmer, personal communication). When making the transitions from patterns starting with a rest, he describes a significant shift in ‘feel’, from playing off the beat to playing on the beat. The patterns that start with a rest were also described as feeling less ‘stable’, the higher cognitive demand leaving less capacity to manage the ‘shift in feel’ when the transition occurs to a pattern starting with an onset. From our empirical data, the four hardest transitions (D, G, I and L) were found to be approximately equally challenging (Fig 12), however there are distinguishing factors to consider in professional performance. Firstly, two of them have five claps in a row around the transition (D and L). The rest of the piece only features a maximum of three claps in a row in each of the patterns, so five ‘feels’ wrong and requires particular concentration. Secondly, the other two hardest transitions (G and I) have another unique feature—presentation of two sets of three claps in a row, separated by just one rest. A technique used to memorise the piece is to identify where the three successive claps are in the bar at any one time, so playing two three-clap sets in a row breaks the flow of performance. Further investigation of these effects could be made using differently structured patterns.

We have shown that data collected from a game-based app can provide detailed empirical evidence of how pattern complexity influences ensemble rhythmic performance. There are many advantages to using a game-based app to collect data on such a large scale. In particular, we were fortunate that the app was successful and so we were able to collect such large datasets from a wide range of participants. However, there were also disadvantages. Large datasets from Internet sources are often unreliable, prone to outages and losses; this requires a thorough understanding of the properties and limits of a dataset, regardless of its size [41]. Factors that made the game so compelling also required us to sacrifice some elements of experimental control. For example, it was important that the composer and the publishers of Clapping Music endorsed the project. A reasonable condition of this was that the game should match a performance as closely as possible. The patterns had to be presented in the order indicated by the score and could not be presented in randomly assigned order. This presented the problem that many players only reached the early patterns, and necessitated the analysis of a restricted subset of the data, completed games, to ensure we treated the patterns equally. However, the very large sample size compensated for this restriction, providing sufficient data for robust analysis of the patterns and transitions that players found most challenging to perform. A further question that can be answered using the data collected is whether this is influenced by individual factors such as age and musical training; this will be addressed in future research.

There have been over 140,000 downloads of the app worldwide and we collected gameplay data from over 102,000 individual device registrations during the period of a year for the present analysis. The data was collected at a detailed level (each individual pattern row tapped by every user), the number of records being in excess of 46 million, requiring high performance big data computing solutions to analyse the data. This is a relatively new research method, especially applied to the investigation of psychological phenomena. However many of the most important sources of big data are also relatively new [42]. For example, the huge amounts of information available from social networks are only as old as the networks themselves; Facebook was launched in 2004, Twitter in 2006. The same holds for smartphones and the other mobile devices that now provide enormous streams of data tied to people, activities, and locations; the iPhone was only unveiled in 2007, and the iPad in 2010. There is little doubt that large quantities of data are now available, but that is not the defining characteristic of this new data ecosystem [41]. Big Data creates a radical shift in how we think about research and reframes key questions about the constitution of knowledge, the processes of research and how we should engage with information. Taken out of context data can lose meaning and value, and during modelling there is a risk of drawing post-hoc conclusions that do not generalise, constraining the analysis post-hoc to fit a particular kind of model [8]. In this study we have the advantage of having been involved in the development of the App, including design and testing of data collection, and having engaged with the community using the finished product through focus groups [11]. As a result, we were able to test hypotheses that had been conceived before the data was collected and analysed. This approach could be applied successfully to many other phenomena influencing the development of musical skills.

## References

[1] London J. Hearing in time: Psychological aspects of musical meter. Oxford: Oxford University Press; 2012.

[2] Grahn JA, Brett M. Rhythm and Beat Perception in Motor Areas of the Brain. Journal of Cognitive Neuroscience. 2007;19(5):893–906. 10.1162/jocn.2007.19.5.893 17488212

[3] Grahn JA, Rowe JB. Feeling the Beat: Premotor and Striatal Interactions in Musicians and Nonmusicians during Beat Perception. Journal of Neuroscience. 2009;29(23):7540–7548. 10.1523/JNEUROSCI.2018-08.2009 19515922 PMC2702750

[4] Repp BH. Sensorimotor synchronization: a review of the tapping literature. Psychonomic Bulletin & Review. 2005;12(6):969–992. 10.3758/BF03206433 16615317

[5] Repp BH, Su YH. Sensorimotor synchronization: A review of recent research (2006–2012). Psychonomic Bulletin & Review. 2013;20(3):403–452. 10.3758/s13423-012-0371-2 23397235

[6] Henrich J, Heine SJ, Norenzayan A. The weirdest people in the world? The Behavioral and brain sciences. 2010;33(2-3):61–83; discussion 83–135. 10.1017/S0140525X0999152X 20550733

[7] Brown HR, Zeidman P, Smittenaar P, Adams RA, McNab F, Rutledge RB, et al Crowdsourcing for cognitive science—The utility of smartphones. PLoS ONE. 2014;9(7). 10.1371/journal.pone.0100662 PMC4099129 25025865

[8] Griffiths TL. Manifesto for a new (computational) cognitive revolution. Cognition. 2015;135:21–23. 10.1016/j.cognition.2014.11.026 25497482

[9] Song Y, Dixon S, Pearce MT. A survey of music recommendation systems and future perspectives. Proceedings of the 9th International Symposium on Computer Music Modeling and Retrieval. 2012; p. 19–22.

[10] Reich S. Writings on music, 1965-2000. Oxford: Oxford University Press; 2002.

[11] Burke A, Palczynski B, Pearce MT, Duffy S, Martyn A. Steve Reich’s Clapping Music Research & Development Report. London: Nesta Digital R&D Fund for the Arts; 2015.

[12] Tranchant P, Vuvan DT, Peretz I. Keeping the beat: a large sample study of bouncing and clapping to music. PloS one. 2016;11(7):e0160178 10.1371/journal.pone.0160178 27471854 PMC4966945

[13] McAdams S, Bregman A. Hearing musical streams. Computer Music Journal. 1979;3(4):26–60.

[14] Bregman AS. Auditory Scene Analysis: The perceptual organization of sound. Cambridge, MA: MIT Press; 1990.

[15] Reich S. Clapping Music; 1972. Universal Editions.

[16] Lerdahl F, Jackendoff R. A Generative Theory of Tonal Music. Cambridge, MA: MIT Press; 1983.

[17] Thaut MH. Rhythm, music, and the brain: Scientific foundations and clinical applications. vol. 7 London: Routledge; 2005.

[18] Lee C. The perception of metrical structure: Experimental evidence and a model In: Howell P, West R, Cross I, editors. Representing Musical Structure. London: Academic Press; 1991 p. 59–127.

[19] Drake C, Penel A, Bigand E. Tapping in time with mechanically and expressively performed music. Music Perception: An Interdisciplinary Journal. 2000;18(1):1–23. 10.2307/40285899

[20] Hannon EE, Trehub SE. Metrical categories in infancy and adulthood. Psychological Science. 2005;16:48–55. 10.1111/j.0956-7976.2005.00779.x 15660851

[21] Hannon EE, Trehub SE. Tuning in to musical rhythms: infants learn more readily than adults. Proceedings of the National Academy of Sciences of the United States of America. 2005;102(35):12639–12643. 10.1073/pnas.0504254102 16105946 PMC1194930

[22] Hannon EE, Soley G, Ullal S. Familiarity Overrides Complexity in Rhythm Perception: A Cross-Cultural Comparison of American and Turkish Listeners. Journal of Experimental Psychology: Human Perception & Performance. 2012;38(3):543–548. 22352419 10.1037/a0027225

[23] Povel DJ, Essens P. Perception of Temporal Patterns. Music Perception. 1985;2(4):411–440. 10.2307/40285311 3991313

[24] Cameron D, Potter K, Wiggins G, M T Pearce MT. Perception of rhythmic similarity is asymmetrical, and is influenced by musical training, expressive performance, and musical context. Timing and Time Perception. 2017;5:211–227. 10.1163/22134468-00002085

[25] Colannino J, Gómez F, Toussaint GT. Analysis of Emergent Beat-Class Sets in Steve Reich’s “Clapping Music” and the Yoruba Bell Timeline. Perspectives of New Music. 2009;47(1):111–134.

[26] Toussaint GT. A mathematical analysis of African, Brazilian, and Cuban clave rhythms In: Proceedings of BRIDGES: Mathematical Connections in Art, Music and Science; 2002 p. 157–168.

[27] Toussaint GT. The pairwise variability index as a measure of rhythm complexity. Analytical Approaches to World Music. 2013;2:1–42.

[28] Patel AD. Music, Language and the Brain. Oxford: Oxford University Press; 2008.

[29] Patel AD, Daniele JR. An empirical comparison of rhythm in language and music. Cognition. 2003;87(1):B35–B45. 10.1016/S0010-0277(02)00187-7 12499110

[30] Huron D, Ollen J. Agogic Contrast in French and English Themes: Further Support for Patel and Daniele (2003). Music Perception. 2003;21(2):267–271. 10.1525/mp.2003.21.2.267

[31] London J, Jones K. Metrical hierarchies and musical nPVI: A re-analysis of Patel and Daniele. In: Proceedings of the 11th International Conference on Music Perception and Cognition; 2010. p. 379–380.

[32] VanHandel L, Song T. The role of meter in compositional style in 19th century French and German art song. Journal of New Music Research. 2010;39(1):1–11. 10.1080/09298211003642498

[33] Hannon EE. Perceiving speech rhythm in music: Listeners classify instrumental songs according to language of origin. Cognition. 2009;111(3):403–409. 10.1016/j.cognition.2009.03.003 19358985

[34] McGowan RW, Levitt AG. A comparison of rhythm in English dialects and music. Music Perception: An Interdisciplinary Journal. 2011;28(3):307–314. 10.1525/mp.2011.28.3.307

[35] Hansen NC, Sadakata M, Pearce MT. Nonlinear changes in the rhythm of European art music: Quantitative support for historical musicology. Music Perception. 2016;33(4):414–431. 10.1525/mp.2016.33.4.414

[36] Mclachlan N. A Spatial Theory of Rhythmic Resolution. Leonardo Music Journal. 2000;10:61–67. 10.1162/096112100570468

[37] Tones DM. Elements of Ewe music in the music of Steve Reich. University of British Columbia; 2007.

[38] Sethares Wa, Toussaint GT. Expressive Timbre and Timing in Rhythmic Performance: Analysis of Steve Reich’s Clapping Music. Journal of New Music Research. 2014;44(1):11–24.

[39] Haack JK. The Mathematics of Steve Reich’s “Clapping Music”. In: Sarhangi R, editor. Bridges: Mathematical Connections in Art, Music, and Science. Southwestern College, Winfield, Kansas: Bridges Conference; 1998. p. 87–92.

[40] Haack JK. Clapping Music-A Combinatorial Problem. The College Mathematics Journal. 1991;22(3):224–227. 10.2307/2686645

[41] Boyd D, Crawford K. Critical questions for big data: Provocations for a cultural, technological, and scholarly phenomenon. Information, communication & society. 2012;15(5):662–679. 10.1080/1369118X.2012.678878

[42] McAfee A, Brynjolfsson E, Davenport TH, Patil D, Barton D. Big data: the management revolution. Harvard business review. 2012;90(10):60–68. 23074865

